# How to use open-pFind in deep proteomics data analysis?— A protocol for rigorous identification and quantitation of peptides and proteins from mass spectrometry data

**DOI:** 10.52601/bpr.2021.210004

**Published:** 2021-06-30

**Authors:** Guangcan Shao, Yong Cao, Zhenlin Chen, Chao Liu, Shangtong Li, Hao Chi, Meng-Qiu Dong

**Affiliations:** 1 School of Life Sciences, Peking University, Beijing 100871, China; 2 National Institute of Biological Sciences, Beijing, Beijing 102206, China; 3 Tsinghua Institute of Multidisciplinary Biomedical Research, Tsinghua University, Beijing 102206, China; 4 Key Lab of Intelligent Information Processing of Chinese Academy of Sciences (CAS), University of CAS, Institute of Computing Technology, CAS, Beijing 100190, China; 5 University of Chinese Academy of Sciences, Beijing 100049, China; 6 Beijing Advanced Innovation Center for Big Data-Based Precision Medicine, School of Medicine and Engineering, Beihang University, Beijing 100191, China

**Keywords:** Protein identification, Mass spectrometry, Search engine, Open-pFind, Quantitation

## Abstract

High-throughput proteomics based on mass spectrometry (MS) analysis has permeated biomedical science and propelled numerous research projects. pFind 3 is a database search engine for high-speed and in-depth proteomics data analysis. pFind 3 features a swift open search workflow that is adept at uncovering less obvious information such as unexpected modifications or mutations that would have gone unnoticed using a conventional data analysis pipeline. In this protocol, we provide step-by-step instructions to help users mastering various types of data analysis using pFind 3 in conjunction with pParse for data pre-processing and if needed, pQuant for quantitation. This streamlined pParse-pFind-pQuant workflow offers exceptional sensitivity, precision, and speed. It can be easily implemented in any laboratory in need of identifying peptides, proteins, or post-translational modifications, or of quantitation based on ^15^N-labeling, SILAC-labeling, or TMT/iTRAQ labeling.

## INTRODUCTION

### Importance of proteomics

In all species of life, gene functions are mostly carried out by proteins, whose total concentration in the cell amounts to 100–300 mg/mL or 2–4 million proteins per femtoliter (Milo 2013). Identifying and quantitating individual proteins from this extremely complex cellular context is the ultimate inspiration of the proteomics technology, which has matured over the past two decades along with the astonishing development of mass spectrometry (MS) instruments (Aebersold and Mann 2016). To date, more than 7000 protein groups can be identified from 200 ng of total HeLa cell proteins in a 2-h liquid chromatography-tandem mass spectrometry (LC-MS/MS, referred to as LCMS hereafter) experiment (Meier *et al*. 2020), and 1056 proteins from a single cell (Cong *et al*. 2021). A proteomics study nowadays may involve hundreds of samples, whereas at the turn of the century it typically meant identification of dozens of proteins from gel slices or a few hundred proteins from immunoprecipitates. In a nutshell, being a powerful and easily accessible analytical technology, proteomics is now visibly transforming biomedical research with a wide range of applications from routine to cutting edge.

### Importance of high-quality, high-speed data analysis

Data analysis is the last step of a proteomics experiment and needless to say, a critical one. Here data analysis refers to identification and quantitation of peptides, proteins, amino acid modifications from LCMS data. Many software tools have been developed for proteomics data analysis, as discussed in several reviews (Chen *et al*. 2020; Hoopmann and Moritz 2013; Valikangas *et al*. 2018).

All data analysis software aims to improve precision, sensitivity and speed. In big-data proteomics, which is characteristic of today’s research, these three qualities are all critical. False positives (low precision) and false negatives (low sensitivity) could lead to disastrous outcomes, for it may take years of follow-up studies for a researcher to realize that it is a false start. And high-speed data acquisition simply demands high-speed data analysis.

### Why pFind 3?

Published in 2018 in *Nature Biotechnology*, the pFind 3 search engine features a powerful and very useful open search workflow (Chi *et al*. 2018). The pFind 3 open search is a handy tool for identifying unexpected modifications, amino acid mutations, and abnormal protease digestions. Moreover, pFind 3 is precise, sensitive, and fast. Tested on four large-scale proteomics datasets (>70,000 MS2 spectra in each), pFind 3 identified on average 77% of the MS2 spectra at 1% FDR, leading to 37%‒73% more unique peptide identifications than six mainstream non-pFind search engines (Chi *et al*. 2018). Very recently, pFind 3 was used to identify extensive post-translational modifications across 100 species (Muller *et al*. 2020).

Here we list five reasons to use pFind 3: (1) Discover unexpected modification or amino acid mutations at no additional time cost; (2) pFind 3 is precise (no inflation of FDR), sensitive (routinely identifies 60%‒80% of the MS2 spectra in a RAW file), and fast (search time ~20 min on a laptop computer for a 2-h LCMS run of digested HeLa cell lysates); (3) Quick assessment of data quality, to see if something is wrong with sample reduction, alkylation, digestion, or desalting, *etc*.; (4) Seamless integration of identification and quantitation based on either MS1 or MS2; (5) Free and friendly.

### The focus of this protocol

In this protocol, we demonstrate proteomics data analysis using the pFind computational platform. We focus primarily on identification of peptides, proteins, and modifications, and also on quantitation based on stable isotope labeling. Of the latter, three quantitation strategies are covered in this protocol: metabolic labeling of proteins using ^15^N (Conrads *et al*. 2001), metabolic labeling of proteins using stable isotope labeling using amino acids in cell culture (SILAC) (Ong *et al*. 2002), and isobaric chemical labeling of peptides (Thompson *et al*. 2003). A total of four datasets (supplementary Table S1 and S2) consisting of five RAW files are used in this protocol:

(1) For identification of peptides and proteins, LCMS data of two technical repeats of a HeLa cell lysate sample: “ID_data_HeLa_QE_HF_120min_rep1.raw”; “ID_data_HeLa_QE_HF_120min_rep2.raw”.

(2) For relative quantitation based on ^15^N, a 1∶1 mix of ^14^N- and ^15^N-labeled *C. elegans* lysate sample: “Quant_15Ndata_Celegans_QE_105min.raw”.

(3) For relative quantitation of phosphorylation based on SILAC labeling, a sample of phosphopeptides enriched from a 1:1 mix of light- and heavy-SILAC labeled HeLa cell lysate: “Quant_SILACdata_HeLa_QE_110min.raw”.

(4) For relative quantitation of proteins based on MS2, a human rhabdomyosarcoma (RMS) cell sample labeled with 6-plex tandem mass tag (TMT): “Quant_TMTdata_RMS_QE_105min.raw”.

### About open-pFind, the open search workflow of pFind 3

**Figure 1:**
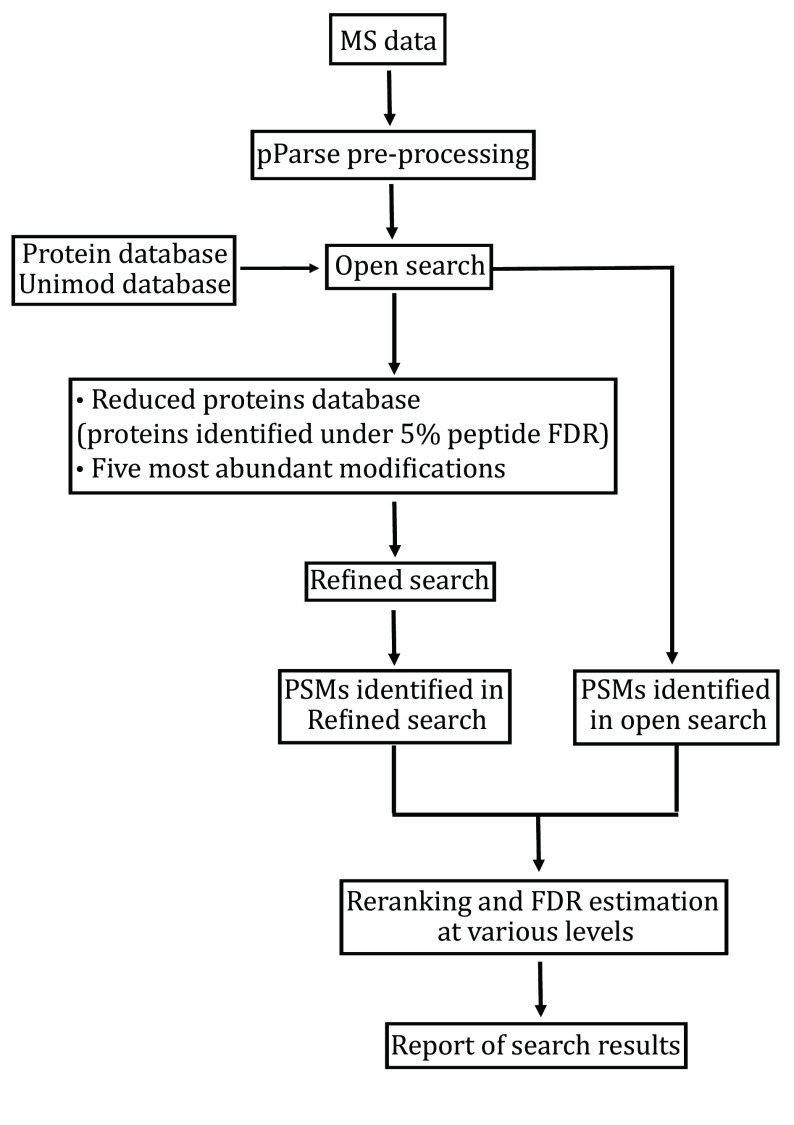
Open-pFind. This workflow of pFind 3 consists of an open search and a refined search. The latter is a generic restricted search performed automatically

The pFind 3 program contains four modules, each for a specific task: pParse for pre-processing of MS data, pFind the core module for database search, pQuant for quantitation, and pBuild for post-processing of search results. Open-pFind, the crown jewel of pFind 3, is a two-step workflow — first an open search and then a refined search — and it involves at least three modules pParse, pFind, and pBuild. Because it is easy to confuse open-pFind with open search, we wish to make clear that they are not interchangeable: the former is the latter plus a refined search. The refined search is a generic, restricted search that is done automatically in open-pFind following the open search. Open-pFind outputs the combined results of the open search and the refined search. Below is a brief description of what happens in an open-pFind search (Fig. 1).

(1) pFind 3 calls on a program called pParse to extract MS1and MS2 peak lists out of the .raw files. pParse looks at the relevant MS1 spectra to verify the monoisotopic *m*/*z* and the charge state of the precursor ion recorded in each MS2 spectrum, and makes corrections as necessary. The precursor *m*/*z* and charge state information in MS2 is recorded on the fly, *i.e.*, while the instrument is performing many tasks simultaneously as required by LCMS analysis. With the benefit of hindsight, pParse makes better judgement. In a particular situation of multiple precursors being isolated and fragmented together, which happens when co-eluting peptides have similar *m*/*z* values, pParse will assign multiple precursor masses to a thus generated multiplexed MS2 spectrum based on the isolation window used and what is found in the neighboring MS1 spectra. In other words, pParse may extract multiple MS2 spectra out of one recorded in the .raw file.

(2) pFind 3 conducts on open search, which means that the protein sequence database is all that it needs from users to search the data that have been pre-processed by pParse. The open search mode of pFind 3 does not need user input for enzyme specificity or modification, the refined or restricted search does. Using sequence tags found in MS2 as index, considering all the modifications collected in the Unimod database (Creasy and Cottrell 2004), considering all theoretically possible peptides from a given protein sequence database, and allowing one amino acid mutation per peptide, pFind 3 scores every peptide-spectrum match (PSM). This is followed by ranking and reranking of PSMs, controlling FDR, and reporting of identification results in a temporary file (Fig. 1).

(3) pFind 3 conducts a refined search using a reduced protein sequence database. This database contains only proteins supported by peptides identified from the open search under 5% FDR at the peptide level (Fig. 1). The five most abundant modifications found by the open search are considered (Fig. 1), the rest of the possibilities in Unimod are ignored. The user specified parameters including FDR cutoff, mass tolerance, enzyme specificity take effect in this automatically performed restricted search.

(4) The PSMs identified in both the open search and the refined search are combined. After reranking, assigning peptides to proteins, and controlling FDR at the spectrum, peptide, and protein levels, pFind 3 outputs the search results (Fig. 1).

## SETUP

### Hardware and software requirement

(1) A personal computer (PC) with at least 2 GB of RAM.

(2) 64-bit version of Microsoft Windows 7 or a newer operating system.

(3) .NET framework 4.5 or a higher version. Available at https://www.microsoft.com/zh-cn/download/details.aspx?id=30653.

(4) 64-bit version of JAVA 8.0 or newer (Windows), for protein quantitation analysis. Available at https://javadl.oracle.com/webapps/download/AutoDL?BundleId=243737_61ae65e088624f5aaa0b1d2d801acb16.

(5) pFind 3, version 3.1.6. Free download at http://pfind.ict.ac.cn/software/pFind/index.html.

(6) Python 3.6 or higher, required for comparing pFind 3 search results. Free download at https://www.python.org/downloads/release/python-366/. ‘Numpy’ package is required for calculating ^15^N labeling efficiency, install it using the following command: pip install numpy.

(7) The python scripts used in this protocol can be download from https://github.com/daheitu/scripts_for_pFind3_protocol.io.

### Installation of pFind 3

Double click on the installation package, choose the language and directory. pFind 3 will then finish the installation automatically. The pFind 3 installation package includes pParse for MS data pre-processing tool, pQuant for quantitation, and pBuild for visualization of results.

[**CRITICAL STEP**] License is required to run pFind 3 at the first time. To receive the license file, send an e-mail to pfind@ict.ac.cn. Follow instructions during installation.

## PROTOCOL

### About the construction of this protocol

**Figure 2:**
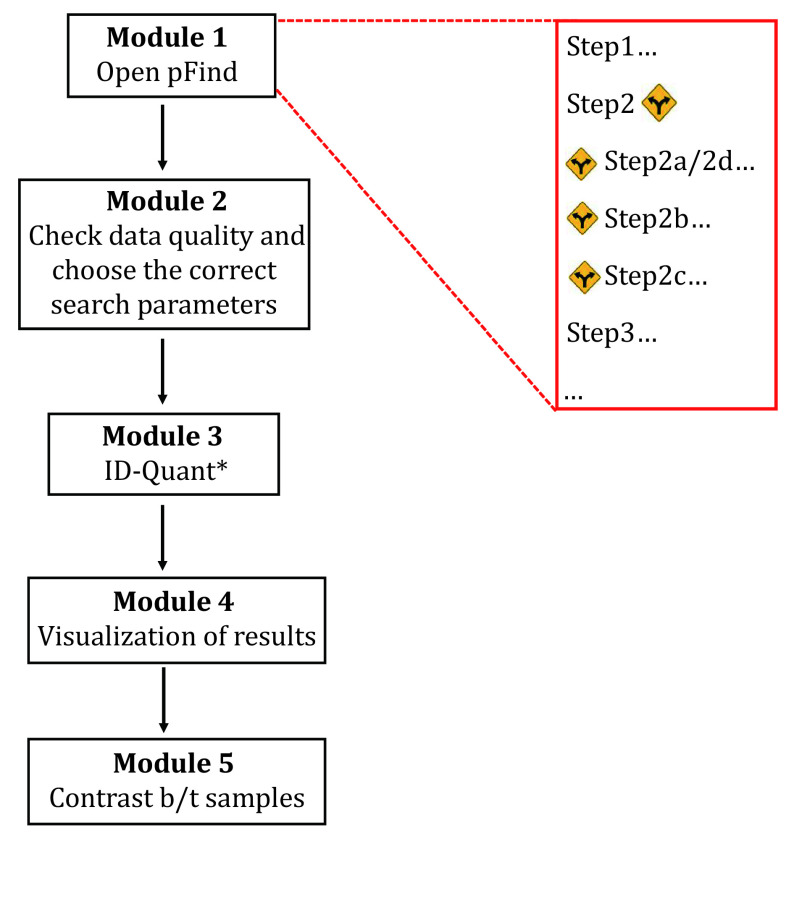
Overview of the proteomics data analysis protocol using pFind 3. *Quantitation is optional.

As illustrated in Fig. 2, this protocol is made up of five modules.

Module 1: Open-pFind, to find the expected and the unexpected modifications;

Module 2: Check & Choose, check data quality and choose suitable parameters;

Module 3: ID-Quant, restricted search for identification and if needed, quantitation;

Module 4: Result visualization, easy navigation with pBuild from spectrum to protein;

Module 5: Contrast, compare the search results of different samples.

In each module, users will find step-by-step instructions facilitated by annotated screenshots of the pFind 3 user interface. At some of the steps, a forked-road sign “

” is in place to indicate a procedural difference for the different purposes of data analysis as listed below.



 a: identification of peptides, proteins, or PTMs;



 b: quantitation based on ^14^N/^15^N labeling;



 c: quantitation based on SILAC labeling;



 d: quantitation based on TMT or similar isobaric chemical labeling.

### Module 1: Open-pFind (time cost: minutes to hours)

**Figure 3:**
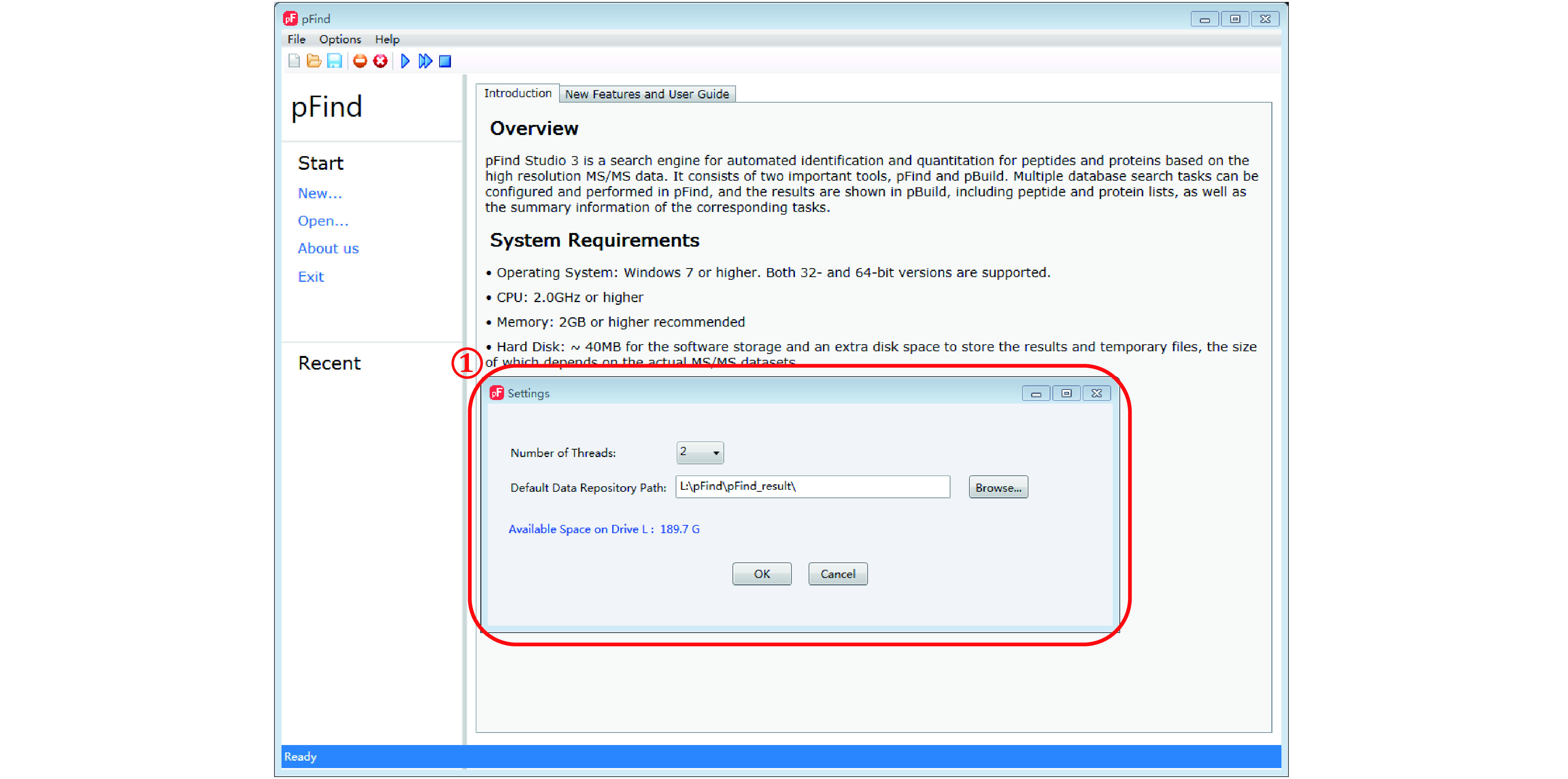
Opening the pFind 3 software for the first time

*Step 1*: *Open the pFind 3 software* (*Fig. 3*)

Double click on the pFind 3 icon on the desktop or the pFind.exe file from the installation folder. When it is opened for the first time, pFind 3 will ask users to select the “Number of Threads” of the computer in use and the “Default Data Repository Path”. The latter is where the search results will be placed. Both can be changed later from the pFind menu under Options/Settings.

**[NOTE]** Leave no space, punctuation marks, or non-English characters in the path. This means that do not name your files or folders in Chinese, Japanese, or Hebrew.

**Figure 4:**
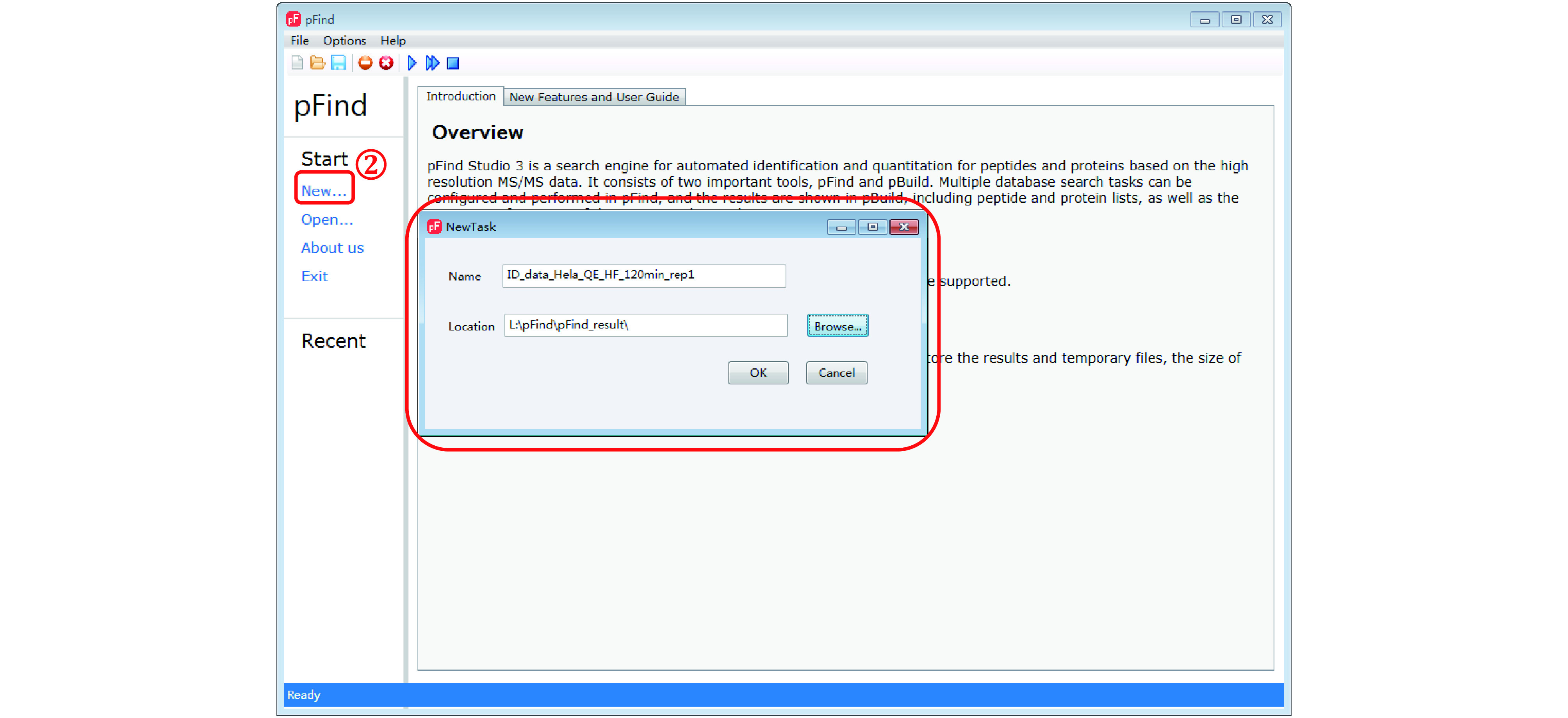
Start a new task

*Step 2*: *Start a new task* (*Fig. 4*)

Click “New…” and in the pop-up window, give the task a “Name” and click on “Browse…” to select a “Location” to place the search result. Click “OK” when finish.

**[NOTE]** Chinese characters are not allowed in the task name and location.

**Figure 5:**
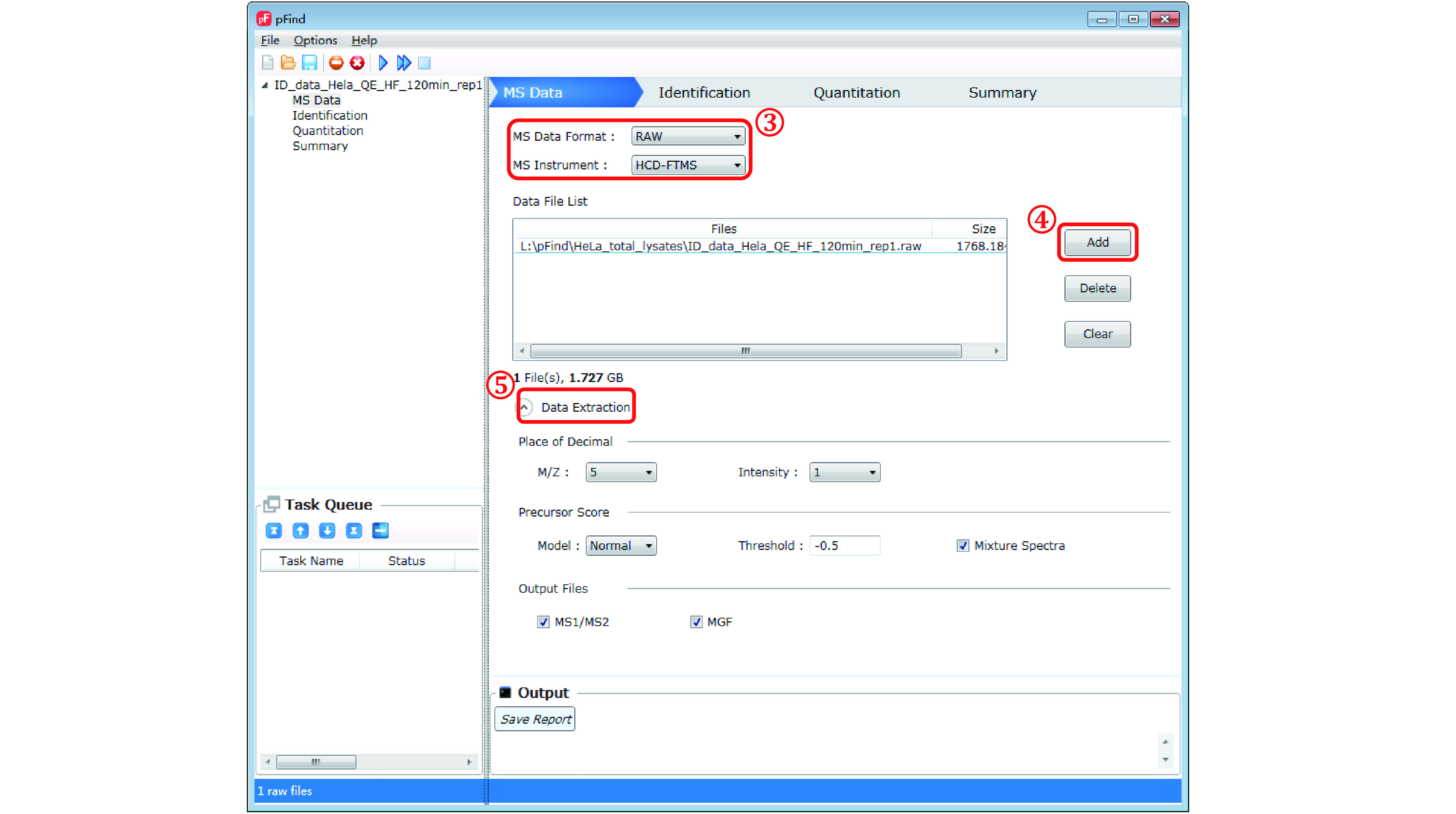
Import.raw files and set up data extraction parameters

*Step 3*: *Describe data type* (*Fig. 5*)

In the “MS Data” panel, specify the “MS Data Format”, for which “RAW” is the default format and four other choices (“MGF”, “RAW”, “WIFF”, “mzML”) are available in the pulldown list. Because all of the sample datasets were acquired on Orbitrap instruments using higher-energy collision dissociation (HCD), “HCD-FTMS” is the right choice for “MS Instrument”. Three other choices are accommodated: “HCD-ITMS”, “CID-FTMS”, and “CID-ITMS”. However, we do not recommend searching ITMS data using the open search mode because the low-resolution data will make the scoring algorithm and the evaluation algorithm much less effective.

**[CRITICAL STEP] **.raw files are highly recommended; .mgf files extracted using other software tools may or may not be supported depending on the details; .ms2 files are not allowed.

*Step 4*: *Load data* (*Fig. 5*)

Click the “Add” button. In the pop-up window select the MS data file(s) to be searched. As an example, for search task *a* — identification of peptides, proteins, or PTMs — one MS file (“ID_data_Hela_QE_HF_120 min_rep1.raw”) is loaded.

*Step 5*: *Data extraction* (*Fig. 5*)

This is typically left to default settings. Click on it to view or hide the details. See supplementary Table S3 for detailed explanation of the related parameters.

**Figure 6:**
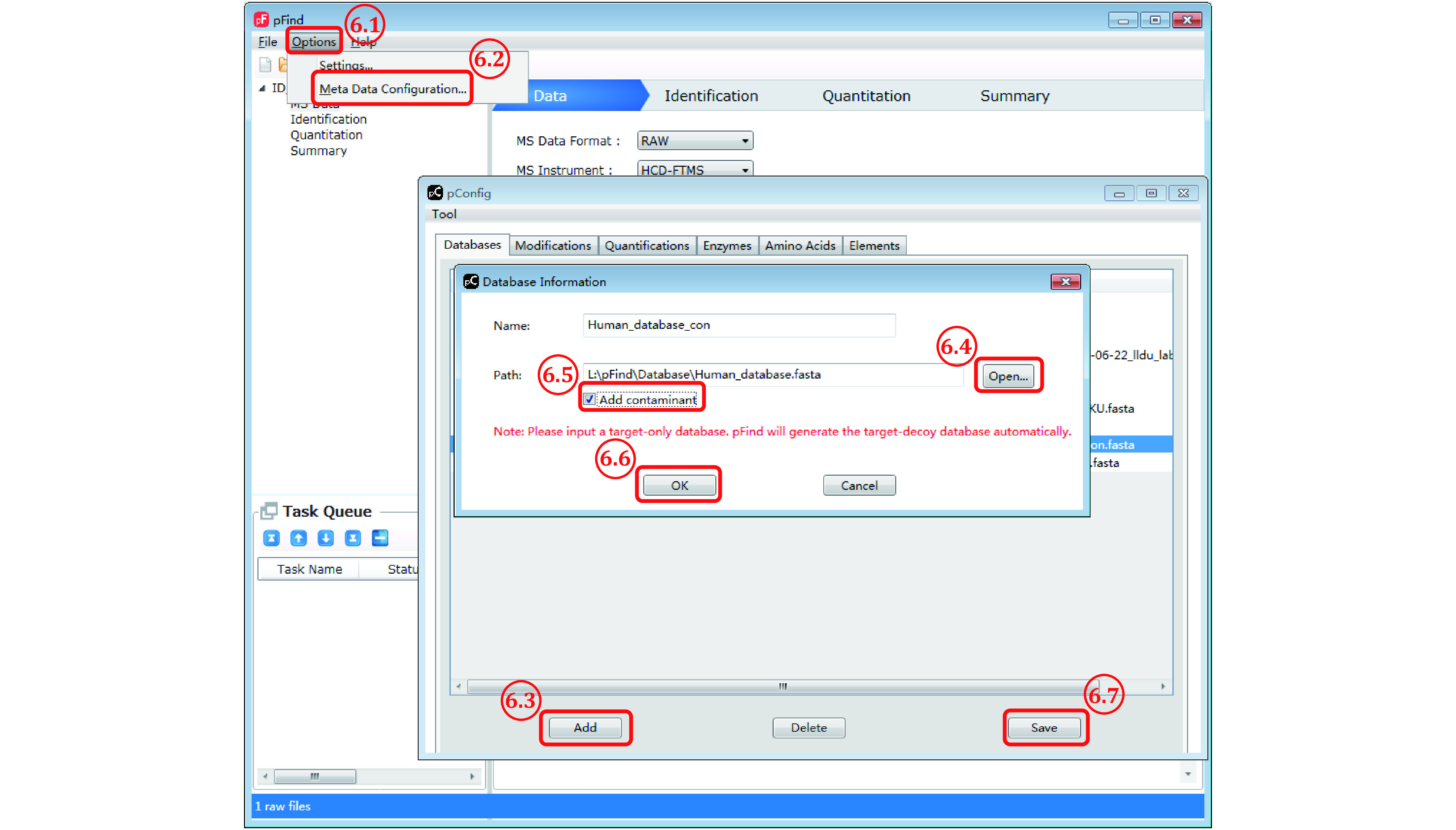
Add a new protein sequence database

*Step 6*: *Add a protein sequence database if it is not in the pFind 3 collection* (*Fig. 6*)

Step 6.1: Select “Options” from the pFind 3 menu bar.

Step 6.2: From the pop-up selection list choose “Meta Data Configuration” and the “pConfig” box will appear.

Step 6.3: Click “Add” to pop up the “Database Information” box.

Step 6.4: In this “Database Information” box, click “Open” to select a desired protein sequence database in the.fasta file format, assuming that it has been downloaded to a local drive. Do not include a database of reversed protein sequences in the original.fasta file because this decoy database will be generated by pFind 3 automatically.

Step 6.5: Check the box of “Add contaminant” if the.fasta file to be loaded does not contain the sequences of common contaminant proteins. pFind 3 will then add to the database its collection of 286 common contaminant proteins, including various keratins from human hair or skin and proteases used in sample digestion, to the original database specified by users.

Step 6.6: Click “OK”.

Step 6.7: Click “Save” to finish loading the new database to the local collection.

**[CAUTION]** Do not use a database containing less than ten proteins because it will result in too small a decoy database and thus erratic FDR estimation, in which case, “Add contaminant” is a necessity.

**Figure 7:**
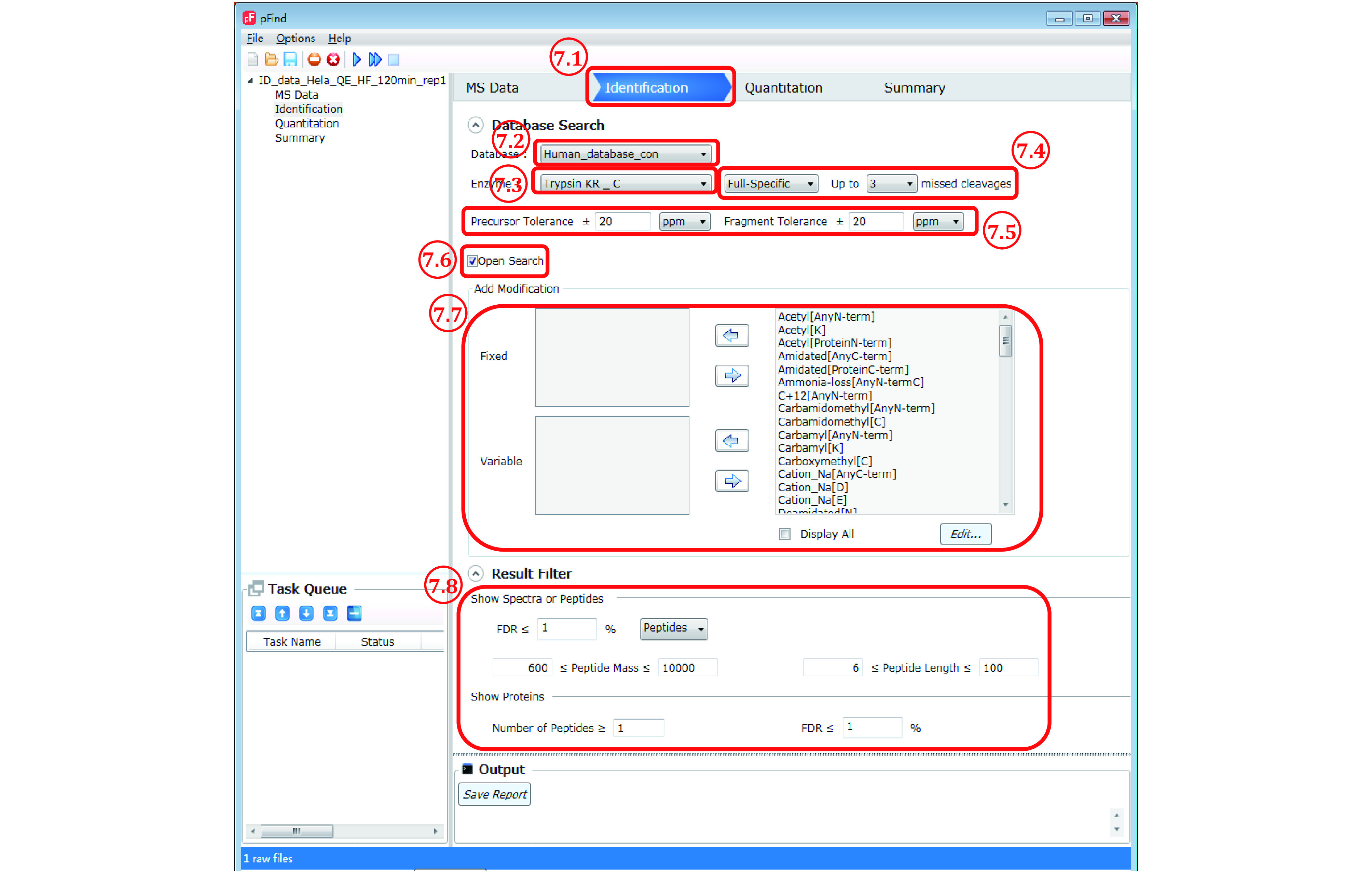
The “Identification” panel

*Step 7*: *Set up identification parameters* (*Fig. 7*)

Step 7.1: Switch to the “Identification” panel.

Step 7.2: Choose an appropriate database to use from the pulldown list, which should include the database added in Step 6.

Step 7.3: Select the enzyme(s) that had been used to digest the protein sample. For trypsin digestion, choose “Trypsin KR_C”, which means that a peptide bond C-terminal to lysine (K) or arginine (R) will cut *in silico*. The alternative choice “Trypsin_P KR P C” is almost the same except that there will be no cut when K/R is followed by a proline (P). Both are approximation of trypsin digestion, which cuts after K/R, and although it does not cut between Kand P, it does between R and P (Huesgen *et al*. 2015).

**[NOTE]** In case of a need for a new enzyme or a new combination of enzymes, select from the menu bar Options/Meta Data Configuration/Enzymes/Add, then specify the name of enzyme, position of cleavage (N- or C-terminal to a target residue), targeted amino acid residue, and exception if there is any.

Step 7.4: Select how strictly you want pFind 3 to observe enzyme specificity. “Full-Specific” means that *in silico* digestion will be done in accordance with the enzyme specificity on both ends of a peptide, and “Semi-Specific” means that in addition to the former, peptides with enzyme specificity observed on one but not both ends are also included. “Non-Specific” means that enzyme specificity is to be ignored completely, and this will cost more time in the restricted search mode. In general, either of the first two choices and up to three missed cleavage sites are recommended, which means that up to three internal K or R residues are allowed for a tryptic peptide.

Step 7.5: Specify “Precursor Tolerance” and “Fragment Tolerance”. Leave them to the default setting of 20 ppm for HCD data acquired on orbitrap instruments.

**[NOTE]** These settings obviously depend on the resolution and accuracy of the mass analyzer used. For ion trap MS2 spectra, “Fragment Tolerance” should be set to ±0.2−0.3 Da. We recommend 20 ppm for “Precursor Tolerance” even for high-resolution MS1 data (*R* > 60000 at 200 *m*/*z*), because for precursors of high *m*/*z* or low abundance the mass deviation can be greater than 10 ppm.

Step 7.6: Select “Open search”. This option is turned on by default.

Step 7.7: “Add Modifications” can be ignored because the open search mode is turned on. Of course, you could add variable modifications of interest, and pFind 3 will give them priority.

Step 7.8: Set “Result Filter” parameters. You may leave them to default values for the initial search and play with them later when you know more of your data.

**[NOTE]** You may filter the results in a highly sophisticated way to suit the purpose of your experiment. See supplementary Table S4 for detailed explanation of these parameters.

*Step 8* [*Optional*] 

**Figure 8:**
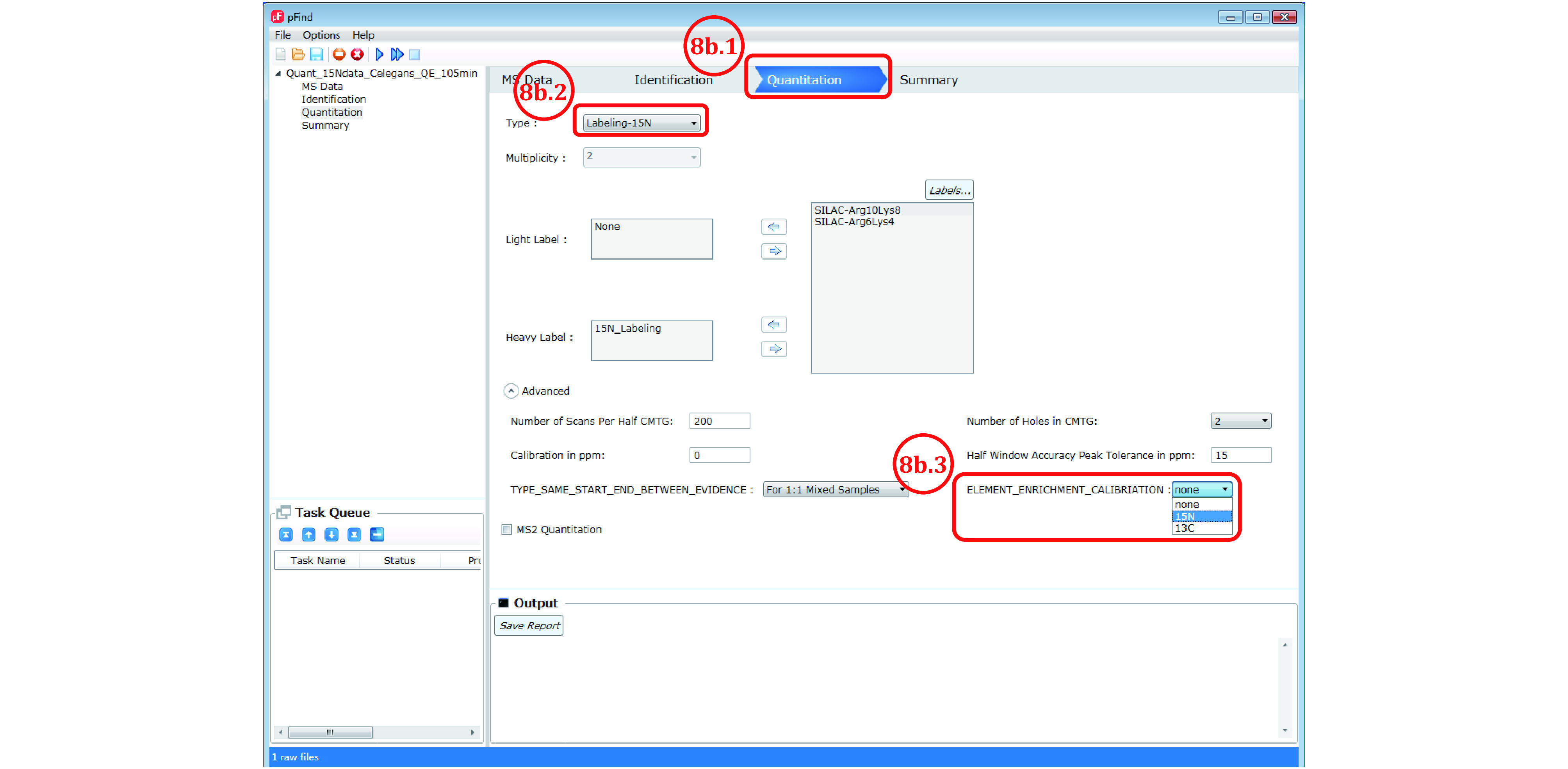
Set the quantitation parameters to obtain 15N atomic enrichment ratios

: *Set quantitation parameters* (*Fig. 8*)

The purpose of this step is to check, when in doubt, the labeling efficiency of the heavy isotope in a 1:1 ^14^N/^15^N sample. This does not apply to SILAC, TMT or iTRAQ. It is not possible to estimate the labeling efficiency of heavy SILAC in a 1:1 light/heavy sample. Instead, the heavy SILAC sample alone needs to be analyzed by LCMS to see what fraction of peptides are left unlabeled. The labeling efficiency of TMT or iTRAQ on the whole can be estimated based on the fraction of unlabeled peptides from the peptide identification result.

No action is taken for the following purposes: identification only, SILAC quantitation, or quantitation by TMT/iTRAQ. For quantitation based on ^15^N labeling (Step 8b 

), follow the steps below to estimate the ^15^N labeling efficiency.



 Step 8b.1: Switch to the “Quantitation” panel (Fig. 8);



 Step 8b.2: Choose “Labeling-15N”;



 Step 8b.3: For “ELEMENT_ENRICHMENT_CALIBRIATION”, select “15N” from the pulldown list, which will instruct pQuant to report the ^15^N labeling efficiency for every heavy isotope-labeled peptide.

**Figure 9:**
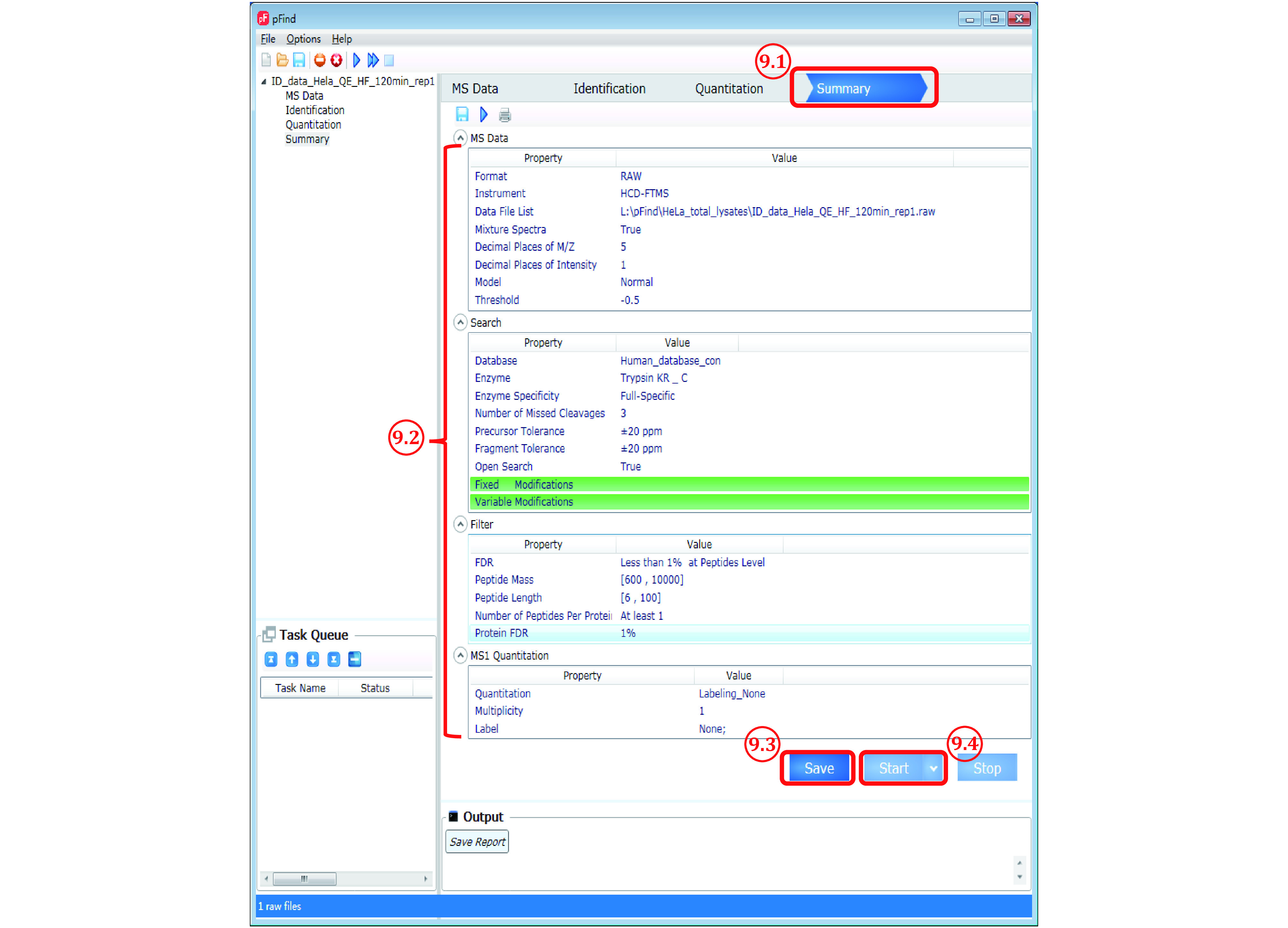
The “Summary” panel of pFind 3

*Step 9*: *Double check all the parameter settings and start search* (*Fig. 9*)

Step 9.1: Switch to the “Summary” panel.

Step 9.2: Double check the parameters. Go back and reset it if something is wrong.

Step 9.3: After verifying the parameters, click Save to activate the “Start” button.

Step 9.4: Click Start and now the search starts.

**[? TROUBLE SHOOTING]**

### Module 2: Check & Choose (time cost: minutes)

In this module we discuss how to check data quality and how to choose suitable parameters to optimize data analysis. After each search, pFind outputs a summary of the search result. It is meant to facilitate proteomics researchers to know the conditions of their data, and then decide what to do next. For example, if the data quality is less than satisfactory, how to improve it? Calibrate the MS instrument and collect the data once more? Further desalting? Redo the digestion using a fresh tube of trypsin? Or start over with a fresh sample? If data quality is good enough, how might the search parameters be adjusted to get the most out of the data? In any case, a careful reading of the summary of results will help.

**Figure 10:**
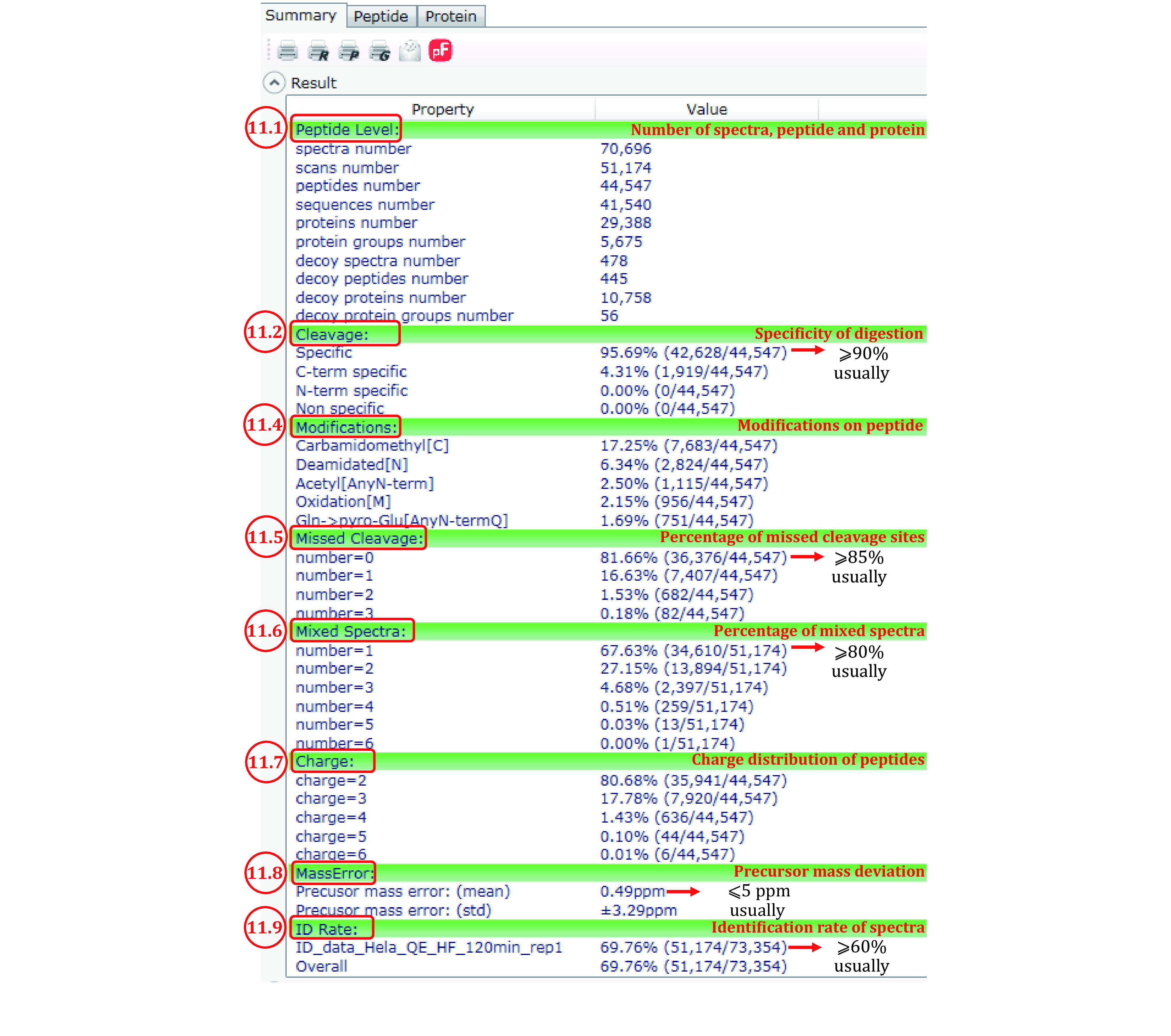
Summary of an Open-pFind search result as displayed by pBuild

*Step 10*: *Open pBuild 3.0 and read the summary of the results* (*Fig. 10*)

pBuild 3.0 is opened automatically to display the results when the search finishes. You can also open pBuild using the desktop shortcut or by clicking on pBuild.exe in the pFind program folder, and then from pBuild open the .tsk file in the result folder. In this module, we focus on the “Summary” panel of pBuild (Fig. 10). See supplementary Table S5 for detailed explanation of the items in the summary page.

*Step 11*: *Check data quality* (*Fig. 10*)

Step 11.1: Numbers of identified MS2 spectra, the corresponding MS2 scans, peptides, sequences (peptides of the same sequence and of different modifications are counted as different peptides but as one sequence), proteins, and protein groups, *etc*. For a cell lysate sample, thousands of protein groups are expected to be identified at 1% protein group FDR.

Step 11.2: Cleavage, or specificity of digestion. For trypsin, expect the percentage of specific digestion (the first one) to be greater than 90%. “C-term specific” means that the peptide C-terminus is generated by trypsin cleavage, but the N-terminus is not. A high percentage of “C-term specific” (*e.g*., 10% or more) and a low percentage of “N-term specific” (*e.g*., no more than 1%) suggest that proteins are cut extensively by additional protease(s) besides trypsin. Most likely, these are proteases found inside the cell. It may be worthwhile to prepare the protein sample one more time and minimize proteolysis during protein extraction. If the percentage of “C-term specific” and that of “N-term specific” are both high, it may be reason to suspect that the trypsin used may have gone bad or severely inhibited for some reasons.

Step 11.3 

: “Quantitation”. Quantitation based on MS1 and stable isotope labeling is summarized here. NaN stands for “Not a Number” and the NaN peptides are those identified only in the light- or heavy-labeled form but not both. For quantitation based on ^15^N labeling, we offer a Python (version 3.6.6) script to obtain the median value of the atomic enrichment ratios of ^15^N-labeled peptides. This is used to check the quality of labeling. Skip this one for identification, SILAC quantitation, or quantitation by TMT/iTRAQ. For ^15^N-labeling (Step 11.3b 

), do the following.



 Step 11.3b.1: Copy the script “Evaluation_of_15N_labeling_efficiency.py” (see the SETUP section) to the result folder, that is, where pQuant.spectra is found.



 Step 11.3b.2: Open “Windows Explorer”, type “cmd” in the location bar, and press “Enter” to open “Command Prompt”.



 Step 11.3b.3: Enter “python Evaluation_of_15N_labeling_efficiency.py” in the command line.



 Step 11.3b.4: The resulting file “Evaluation of 15N labeling efficiency.txt” reports the total number of ^15^N-peptides along with the range, middle 50, median, and mean of ^15^N atomic enrichment ratios. If the median ^15^N atomic enrichment ratio is 95% or above, then the labeling efficiency of the ^15^N reference sample is “OK”. If not, make another and better ^15^N-labeled reference sample.

Step 11.4: “Modification”. The top ten most abundant modifications are listed here. “Expect Carbamidomethyl[C]” to be the most abundant one (typically 10%−15%) in standard proteomics samples using iodoacetamide to alkylate reduced cysteine residues. Also common are asparagine deamidation and methionine oxidation, denoted by “Deamidated[N]” and “Oxidation[M]”, respectively. However, if exceeding 10%, they may be sounding the alarm that the sample has gone stale. “Gln->pyro-Glu[AnyN-termQ]” and a few other modifications frequently rise to the top ten list, but usually no more than a few percent. The metal ion adducts such as sodium, potassium or calcium adducts are usually below 1%. If you see a lot of them, consider desalting the peptides once more.

Step 11.5: “Missed Cleavage”, another indication of the quality of sample digestion. Normally for tryptic digests, more than 90% of the peptides identified have no missed cleavage sites (number = 0, that is, no internal K or R residues). If this percentage drops below 85%, the sample digestion step likely needs trouble shooting.

Step 11.6: “Mixed Spectra”, an indicator of how well the LC separation matches with the complexity of the sample. If peptides are well separated, the percentage of single-precursor MS2 spectra (number = 1) among all MS2 spectra is expected to be 90% or above. If this percentage falls below 80%, perhaps re-run the sample using a longer LC gradient or a longer analytical column.

Step 11.7: “Charge”, or the charge states of the identified peptides. In conventional proteomics experiments, doubly protonated peptide precursor ions (number = 2) usually dominate (90% or higher).

Step 11.8: “MassError”. The precursor mass errors should be less than 5 ppm for high-resolution data acquired on orbitrap instruments. If higher, it may be time to calibrate the instrument.

Step 11.9: “ID Rate”, the percentage of MS2 spectra identified by Open-pFind among all the MS2 spectra in the searched RAW files. Normally, the ID rates are above 60%, up to 85%. If any of the RAW files has an ID rate well below 60%, something may be wrong with that particular run or fraction.

*Step 12*: *Choose suitable parameters to optimize data analysis*

It is important to know from the Open-pFind search how to set “Modifications” for the follow-up restricted search. We recommend one fixed modification, which is usually “Carbamidomethyl[C]”, with 3−4 variable modifications. In other words, you may take from Step 10.3 the top 4 or top 5 most abundant modifications into account in the next search. Including additional modifications will make the search time longer and the FDR rate of the search result higher.

### Module 3: ID-Quant (time cost: minutes to 2 hours per RAW file)

Based on what we know about the data from the Open-pFind search above, we can now optimize data analysis by setting up a restricted search or a restricted search in conjunction with quantitation.

*Step 13*: *Open the pFind 3 software and start a new task for restricted search*

Repeat Steps 1–5. If you set the location of the current search result the same as that of the Open-pFind search above, pFind 3 will see that the MS data have been extracted and thus will skip this step. However, be aware that if you use the exact same location, the earlier open-pFind search result will be replaced by the upcoming restricted search result.

**Figure 11:**
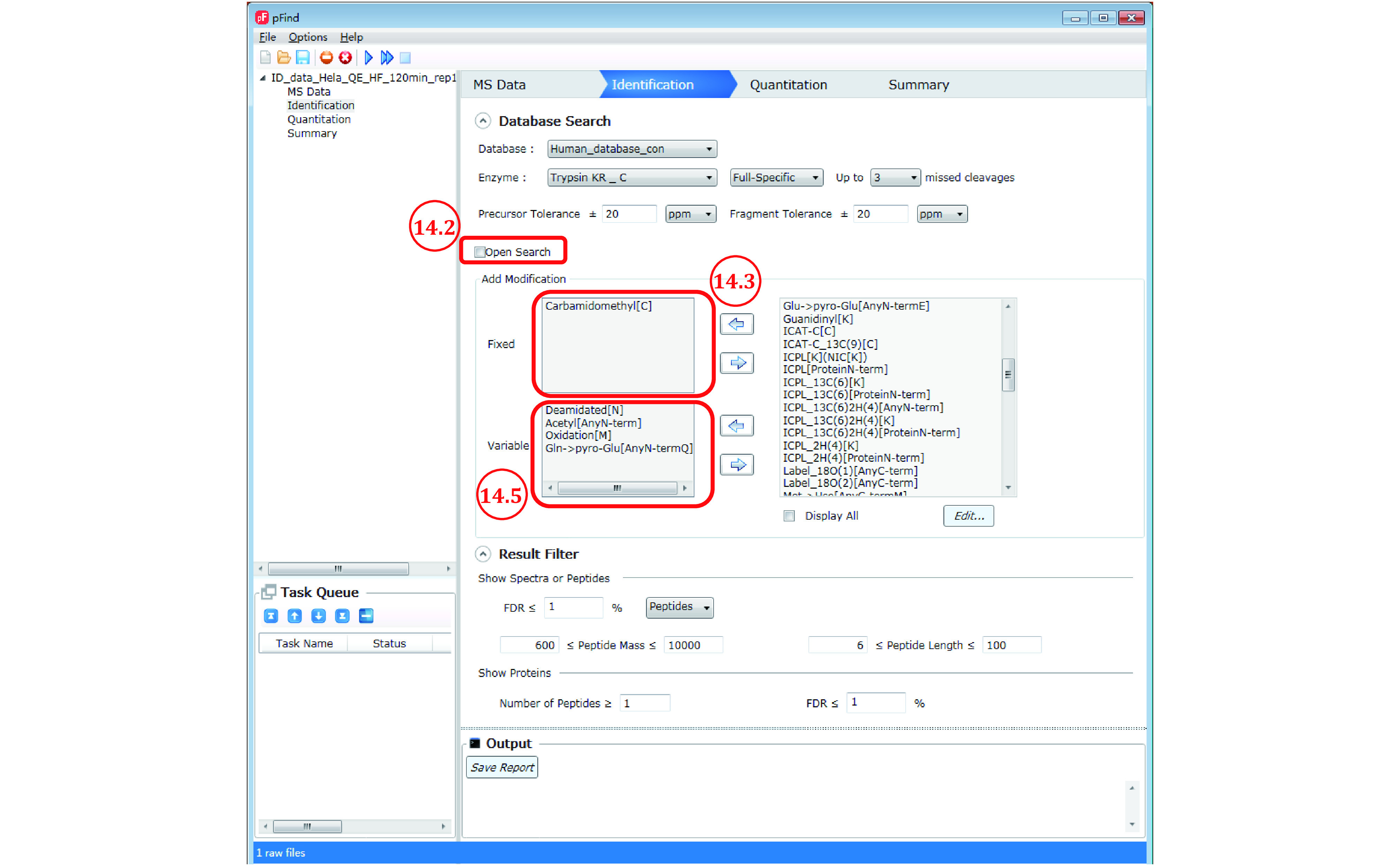
Setting up the identification parameters for restricted search

*Step 14*: *Set up the identification parameters for restricted search* (*Fig. 11*)

Here we restrict the search to the most abundant modifications found by Open-pFind. One fixed modification and 3−4 variable modifications are recommended. More than five variable modifications lead to higher FDR.

Step 14.1: Repeat Steps 7.1–7.5.

Step 14.2: Uncheck “open search”.

Step 14.3: Select “Carbamidomethyl[C]”— the most abundant and nearly complete modification in standard samples — from the modification box on the right and add it to the “Fixed” modification box on the left by clicking the left arrow in between. Of course, if alkylation is far from completion, set it as a variable modification as below or better, redo sample preparation.

Step 14.4 [Optional]: Set a PTM or PTMs of interest in restricted search regardless of frequency of occurrence. Select them one by one from the modification box on the right and add to the “Variable” modification box on the left (Fig. 11) by clicking the left arrow in-between. For phosphorylation for instance, select “Phosphorylation[S]”, “Phosphorylation[T]”, and “Phosphorylation[Y]”.

**[NOTE]**: If you do not find the PTM you want, check the “Display All” option and you will see a full list of PTMs went into the open search. To add a custom modification, see the answer to the frequently asked question (FAQ) No. 7.

Step 14.5: Set up to four of the most abundant modifications from Step 11.4. Select them one by one from the modification box and add to the “Variable” modification box (Fig. 11). Of course, if there is a modification of interest, add it to the search as a variable modification regardless of its abundance.

Step 14.6: Customize the “Result Filter” parameters if unsatisfied with the default setting (see Step 7.8).

*Step 15* 

: *Set Quantitation parameters*



 Step 15a: No action taken if the search is for identification only.



 Step 15b: For quantitation based on ^15^N labeling, see Step 8b.



**Figure 12:**
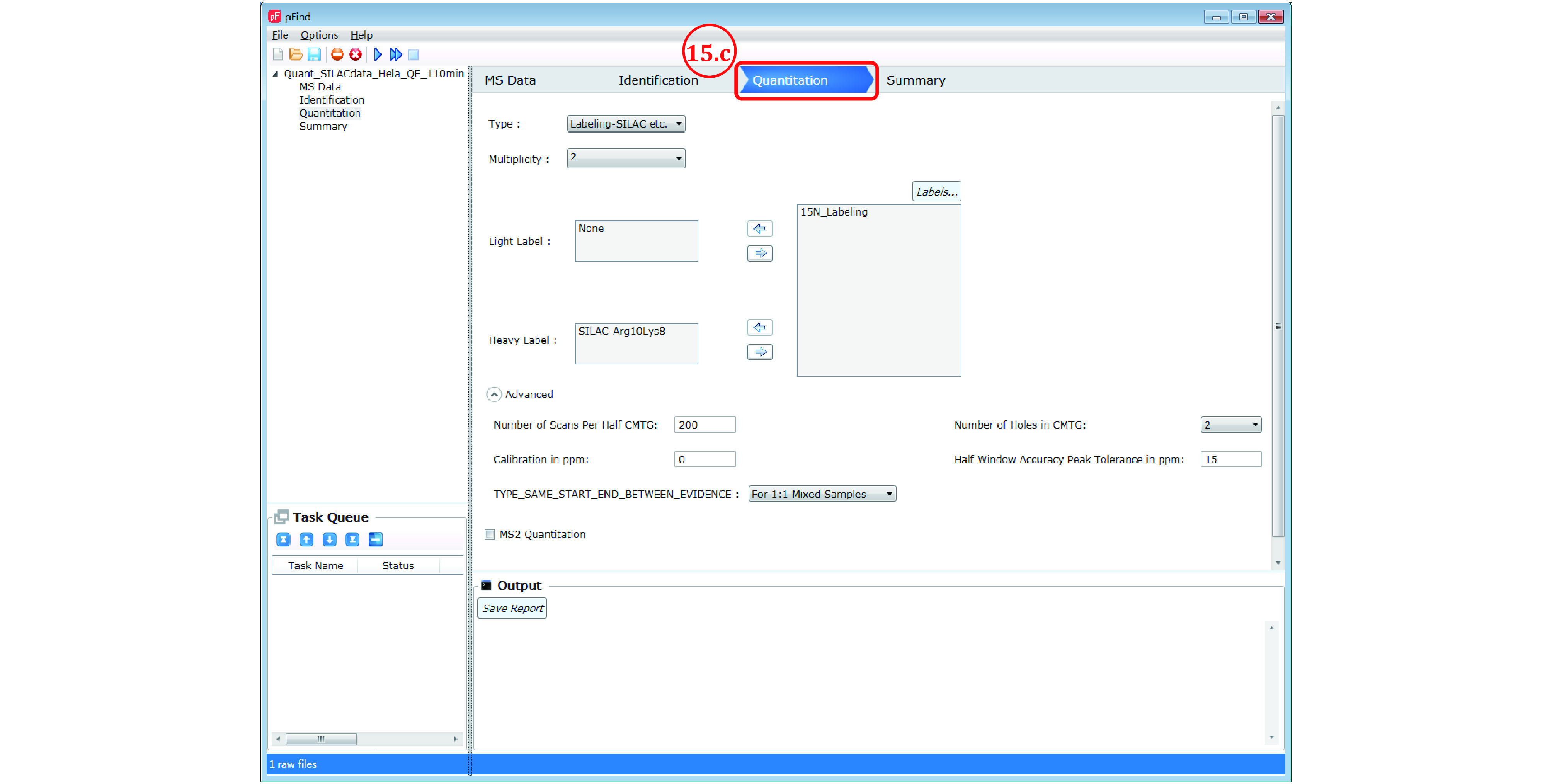
Set quantitation parameters for SILAC data

 Step 15c: For SILAC quantitation, *e.g*., with 1:1 mix of unlabeled (Arg0Lys0) and Arg10Lys8 (Arg-^13^C_6_^15^N_4_, Lys-^13^C_6_^15^N_2_) labeled sample, see Fig. 12. In the quantitation panel select “Labeling-SILAC etc” as the “Type” of quantitation strategy, leave the “Light Label” box as is, select “SILAC-Arg10Lys8” from the “Labels…” box on the right and move to the “Heavy Label” box on the left by clicking the left arrow in-between, and leave the other parameters in default.

**[NOTE]** In the case of triple SILAC labeling, *e.g*., with Arg0Lys0, Arg6Lys4 (Arg-^13^C_6_, Lys-^2^H_4_), and Arg10Lys8, the medium label Arg6Lys4 has not been entered into the “Labels…” box, and thus must be added. Please check out the answer to FAQ No. 8, in which we demonstrate how to add the medium label and set the quantitation parameters.



**Figure 13:**
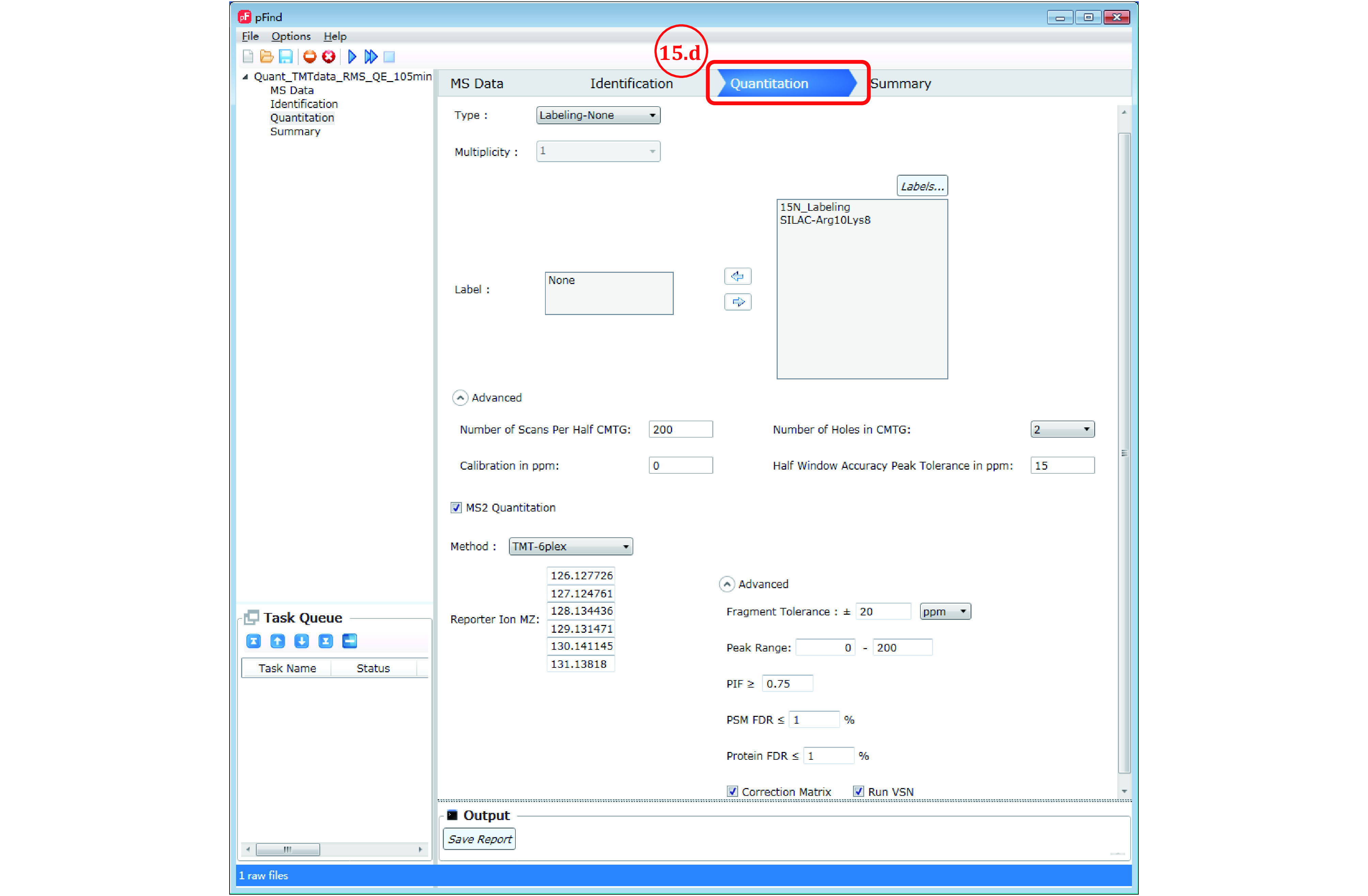
Set quantitation parameters for TMT data

 Step 15d: For TMT data, see Fig. 13. Leave the “Type” of quantitation strategy at its default value of “Labeling-None” and make sure to check “MS2 Quantitation” (the default setting is off). Then, in the “Method” box below, choose the appropriate label. For our example dataset, choose “TMT-6plex”. The other parameters are usually left at the default values. See supplementary Table S6 for detailed explanation of these parameters.

*Step 16*: *Double check all the parameter settings, save, and start searching*

Step 16.1: Switch to the “Summary” panel.

Step 16.2: Double check the parameters. Go back and reset it if something is wrong.

Step 16.3: After verifying the parameters, click Save to activate the “Start” button.

Step 16.4: Click Start and now the search starts.

**[? TROUBLESHOOTING]**

### Module 4: Result visualization (time cost: as long as you wish)

pBuild offers a fantastic user interface for visualizing the ID-Quant results, including identified proteins, peptides, spectra, and chromatograms of light- and heavy-isotope labeled precursor ions. The following is a brief tour of the pBuild features. Much is left for users to discover on their own, especially those related to quantitation. Lastly, we offer two Python scripts to compare results across samples.

*Step 17*: *Open pBuild and check out the summary of the search.*

**Figure 14:**
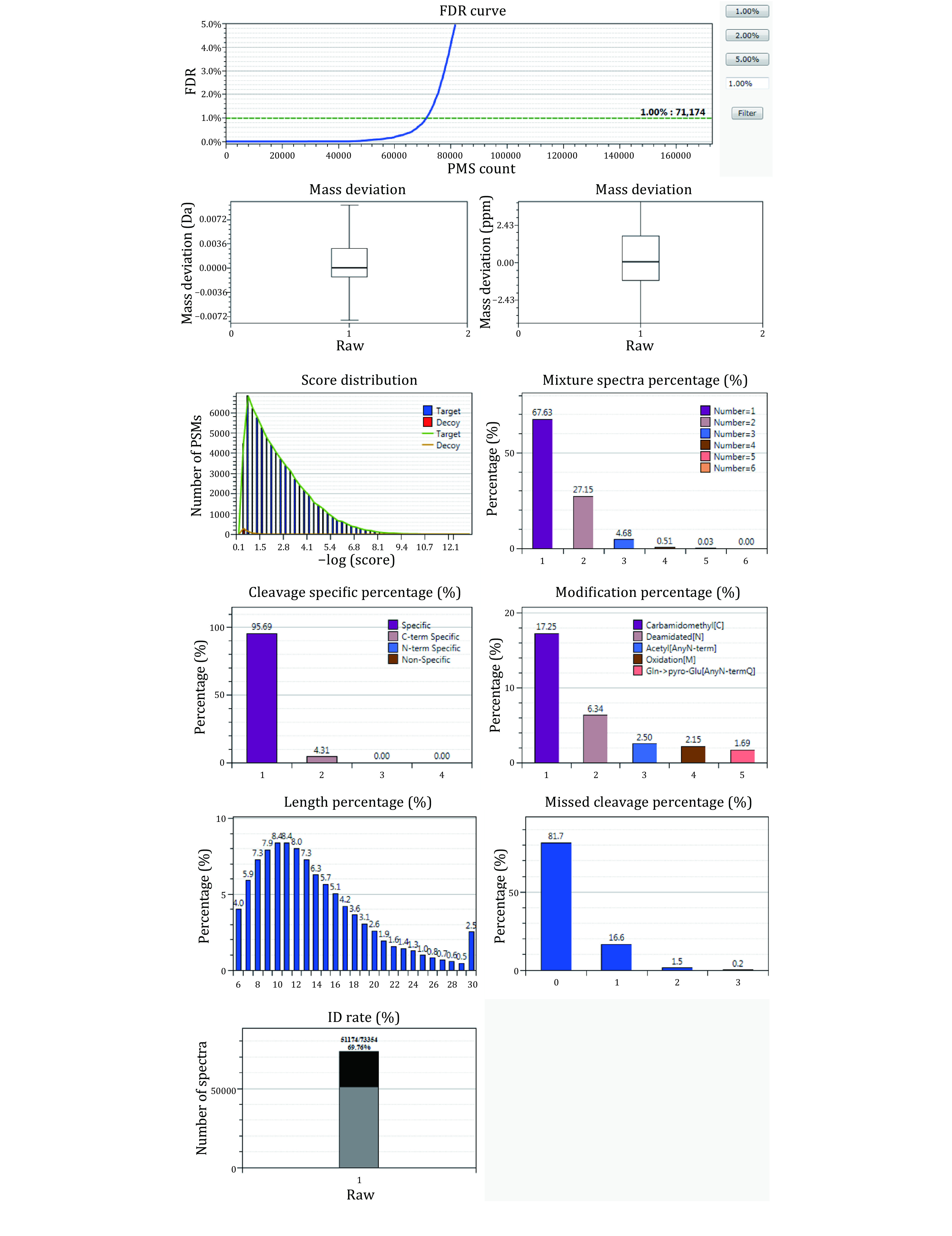
Graphic view of the statistics of search results

Beyond what is shown in Fig. 10 (see Steps 10–11), the summary also displays the statistics of the search result in plots, as shown in Fig. 14.

*Step 18*: *Inspect identified proteins*

**Figure 15:**
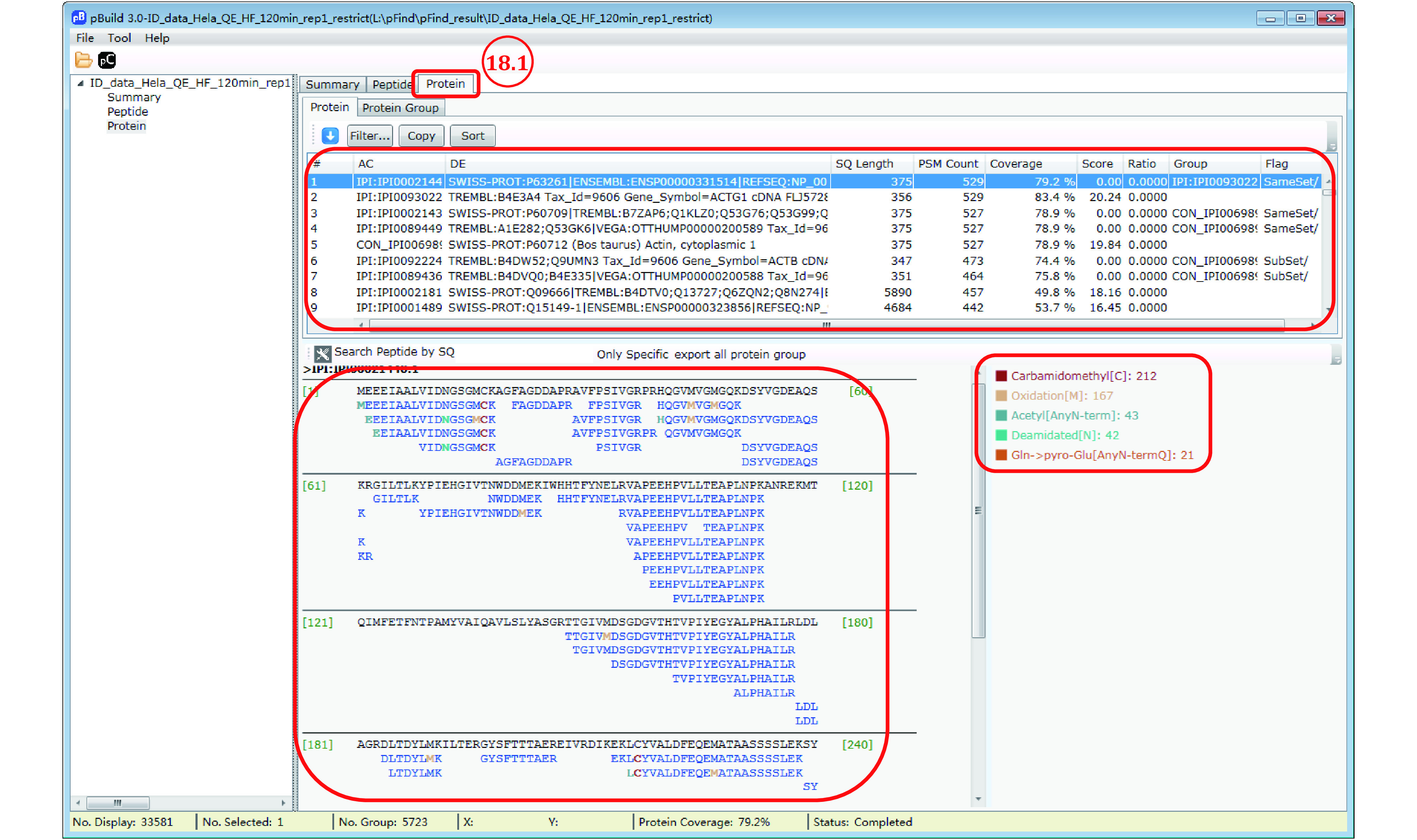
View sequence coverage and modification of proteins

Step 18.1: Switch to the “Protein” panel to access the detailed information of each identified protein, including PSM counts, peptide sequences, sequence coverage, and modifications (Fig. 15).

**Figure 16:**
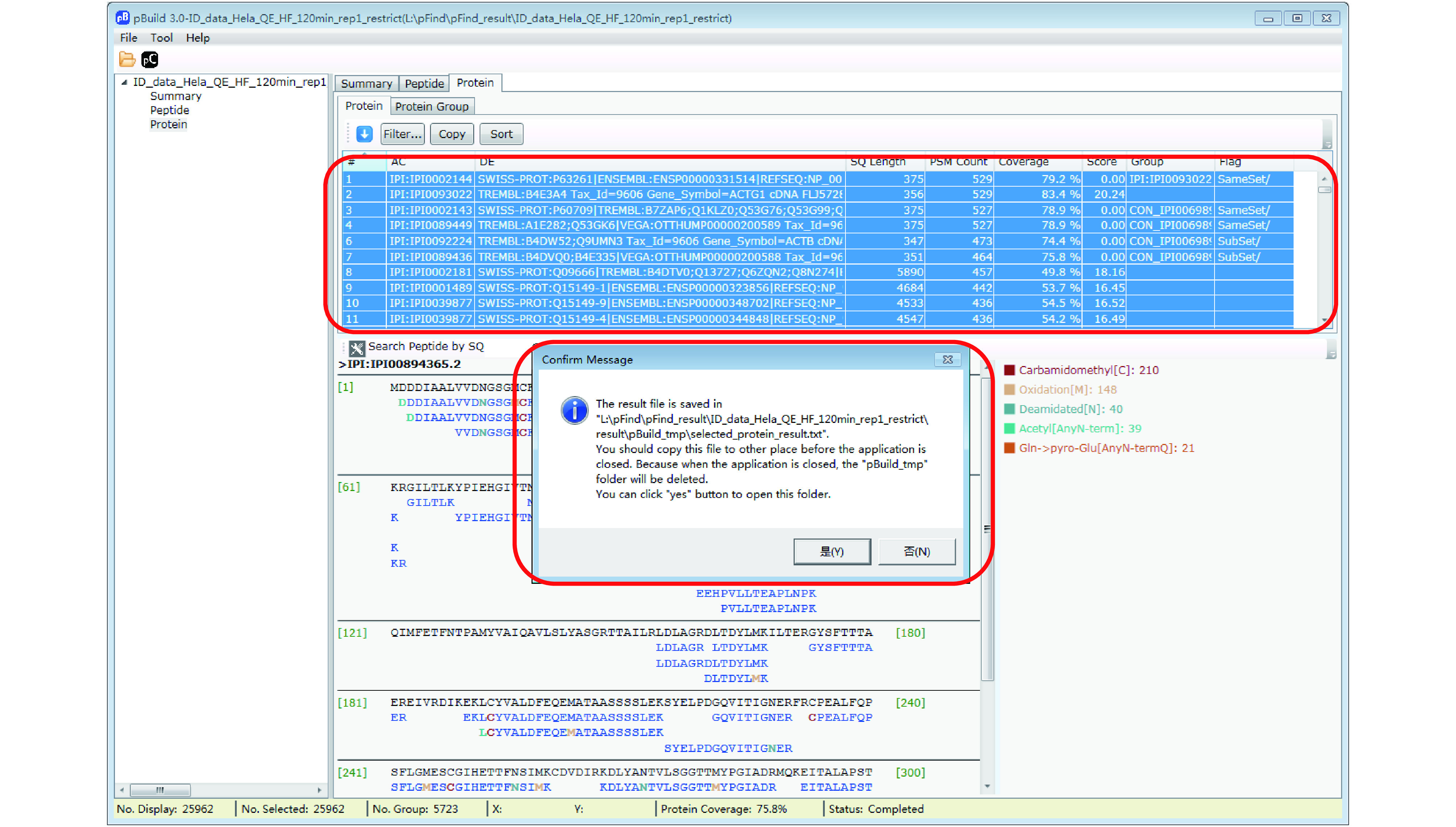
Export the protein identification result

Step 18.2 [Optional]: Export the protein identification results for further inspection. In the “protein” panel, press “Ctrl + A” to select all the result and then press “Ctrl + C” to copy the result, upon which a message box will appear (Fig. 16). Follow the message by copying the saved protein list to a different location. Annotation of this protein identification file can be found in supplementary Table S7.

**Figure 17:**
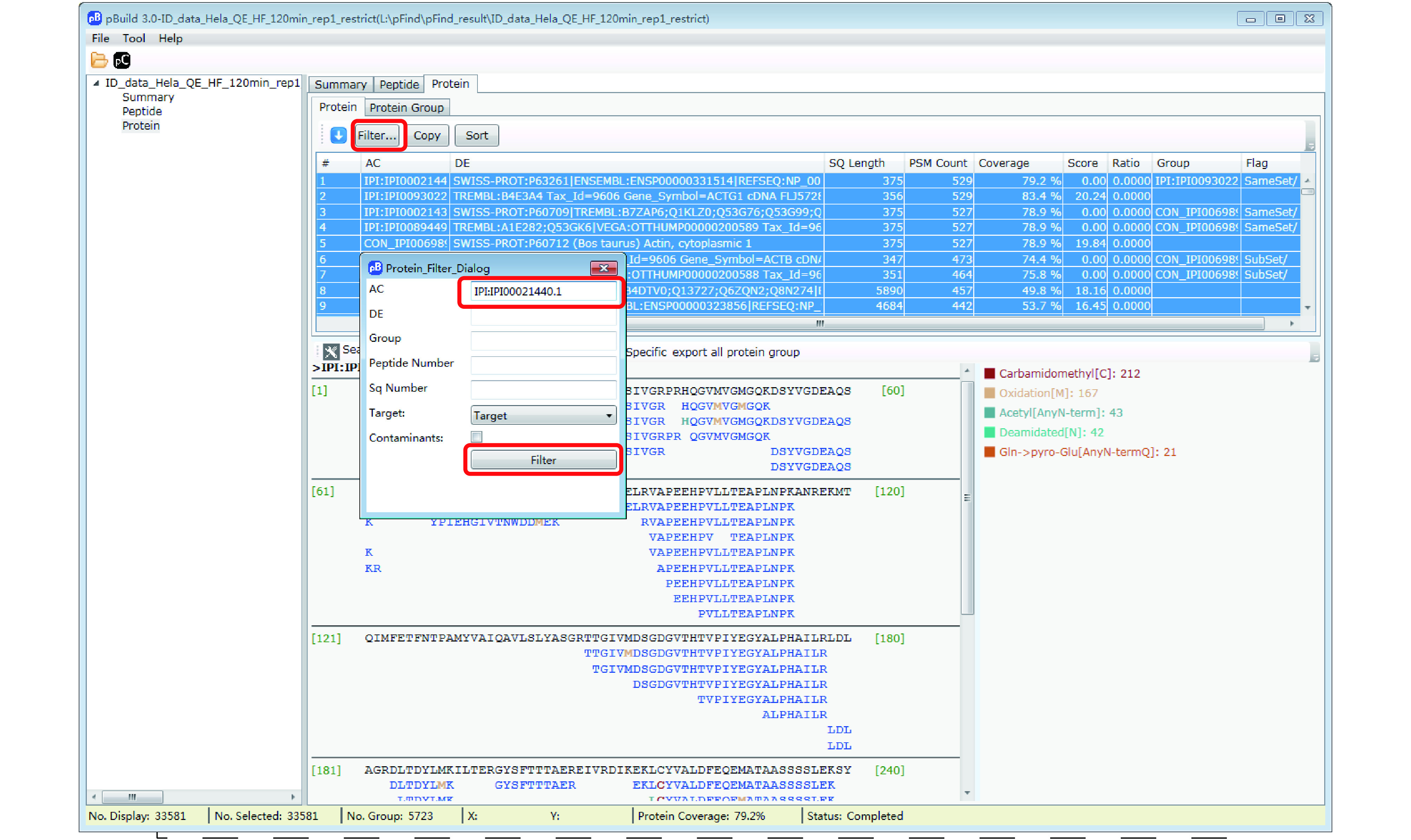
Find a protein of interest

**Figure 18:**
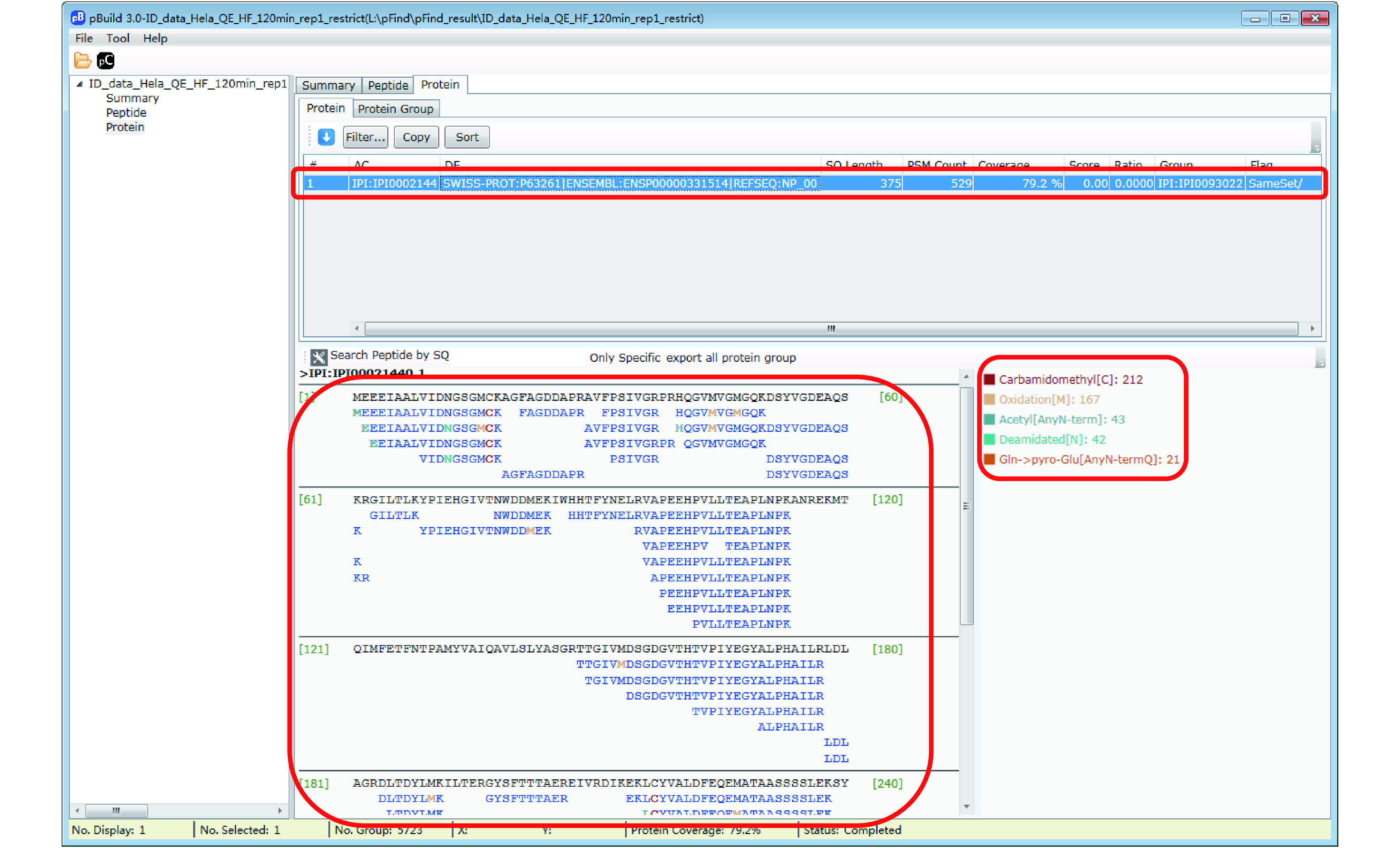
Inspect the peptides and modifications of a protein of interest

**Figure 19:**
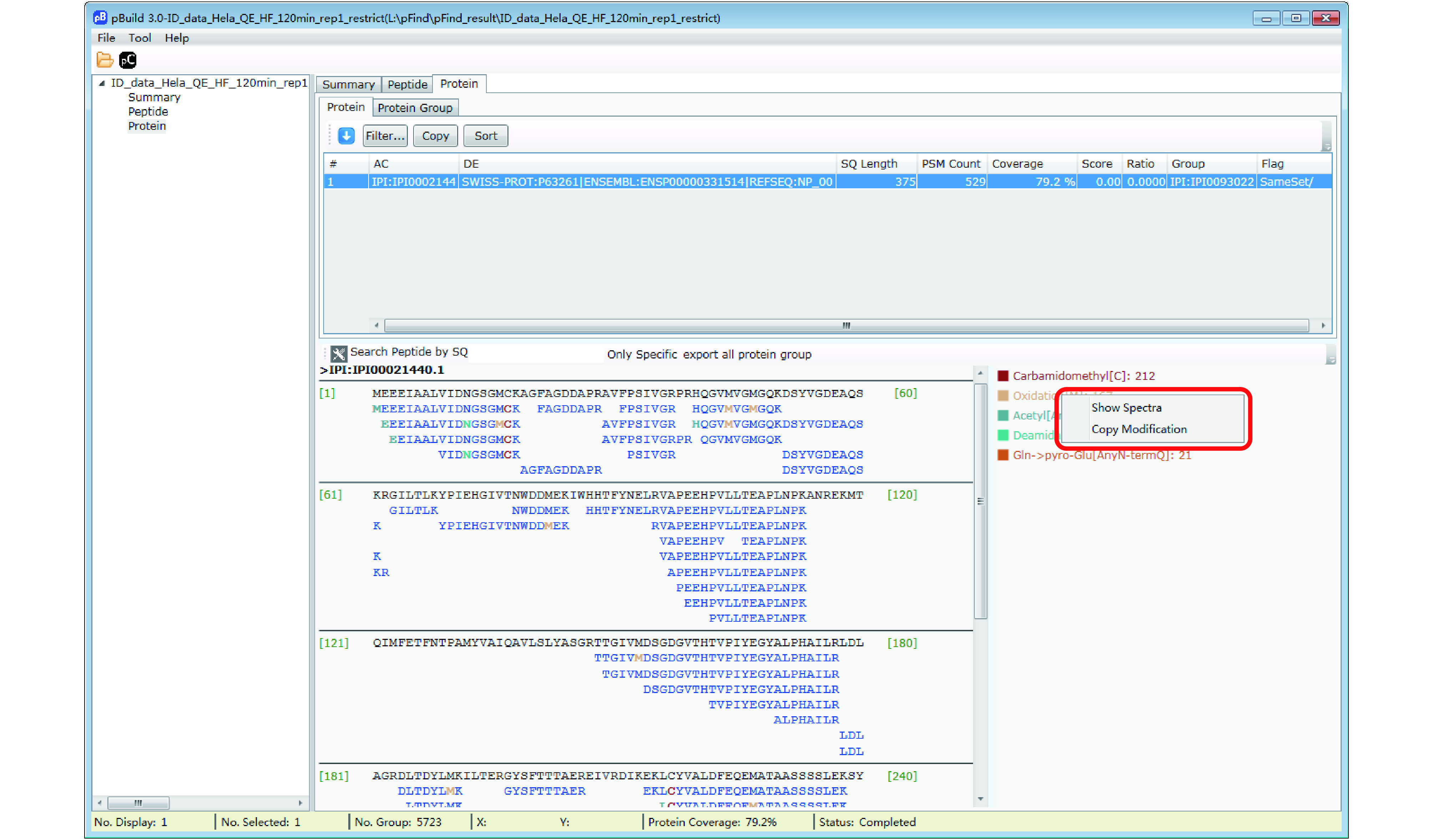
Find the modification spectra

**Figure 20:**
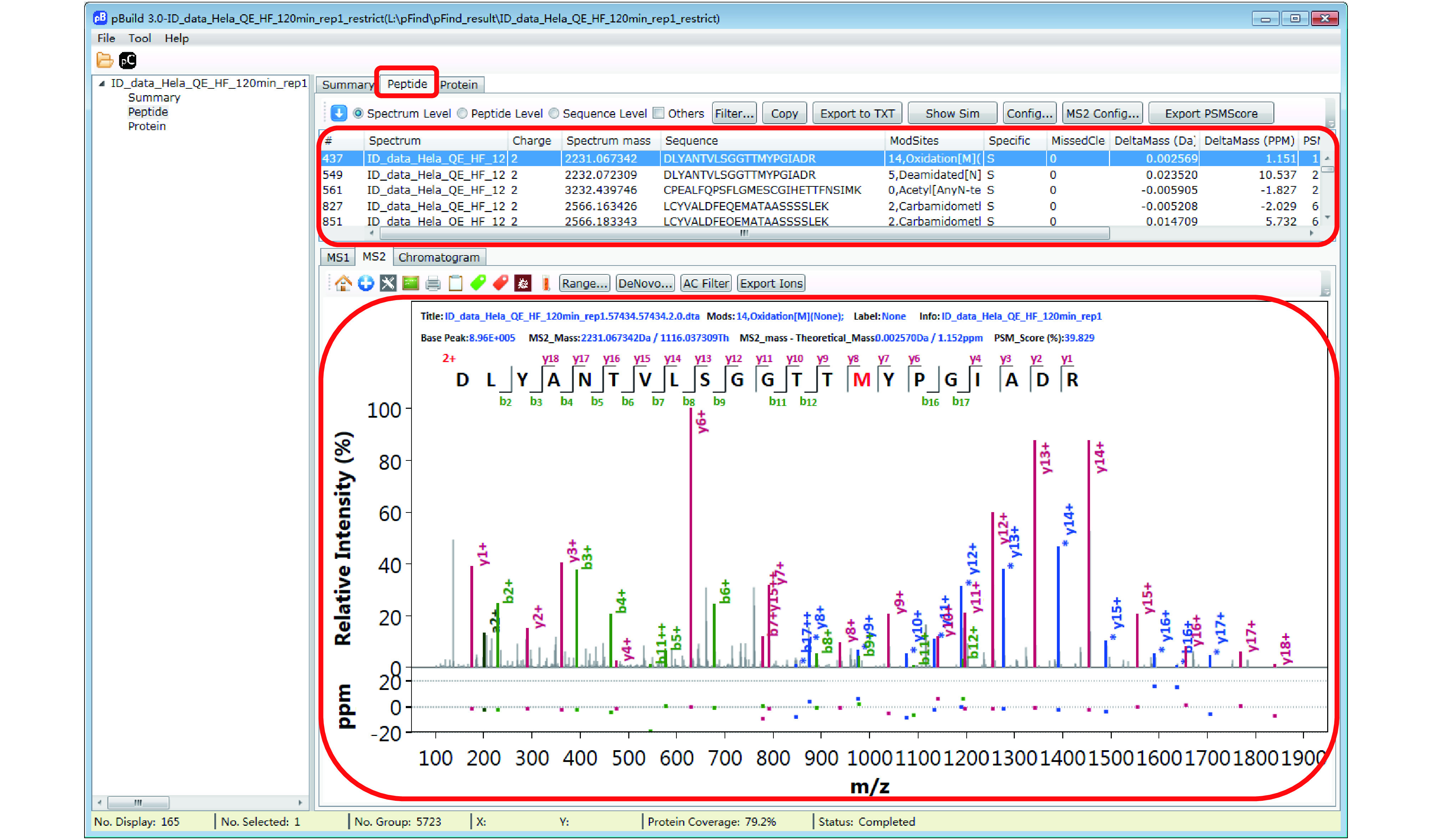
Inspect the modification spectra

Step 18.3 [Optional]: Inspect modifications and the supporting MS2 spectra of a protein of interest (Figs. 17–20). Here we use Oxidation[M] on protein IPI:IPI00021440.1 as an example. As shown in Fig. 17, in the “Protein” panel, click “Filter” to pop up the “Protein_Filter_Dialog” box and enter “IPI:IPI00021440.1” in the field next to “AC” (accession). Click “Filter” and pBuild will display this one protein. The modifications found on this protein are shown on the right side (Fig. 18). Right-click on “Oxidaton[M]” and select “Show Spectra” from the pop-up box (Fig. 19). This will activate the “Peptide” panel in which all the peptides of IPI:IPI00021440.1 that contain an oxidized methionine are now gathered. Click on a peptide to see the annotated spectrum (Fig. 20).

*Step 19* [*Optional*]: *Inspect the identified peptides and associated MS2 spectra*

There are many features in the “Peptide” panel of pBuild (Fig. 20). We encourage users to explore these features independently, freely, and have fun.

### Module 5: Contrast (time cost: minutes)

In this module we offer two Python scripts “pFind_protein_contrast_script.py” and “pFind_PTM_contrast_script.py” (see the SETUP section) to contrast results of different samples or those of different repeats of the same sample. It assists users to quickly find the differences between samples or to check repeatability of LCMS analysis. A user may run these scripts when there is only one dataset, to look at the protein identification result in a simple layout or to extract the PTM of interest.

*Step 20*: *Contrast*

Step 20.1: Create a new folder in a desired location and give it a name.

Step 20.2: To compare across samples either the identified proteins or PTMs, respectively, copy either “pFind_protein_contrast_script.py” or “pFind_PTM_contrast_script.py” and the different pFind.protein files to be compared into this new folder. Note that each pFind.protein file should be renamed before it comes to this folder. The file name should be a ready reminder of the sample and the purpose of the search. Header annotation of pFind.protein file can be found in supplementary Table S8.

**Figure 21:**
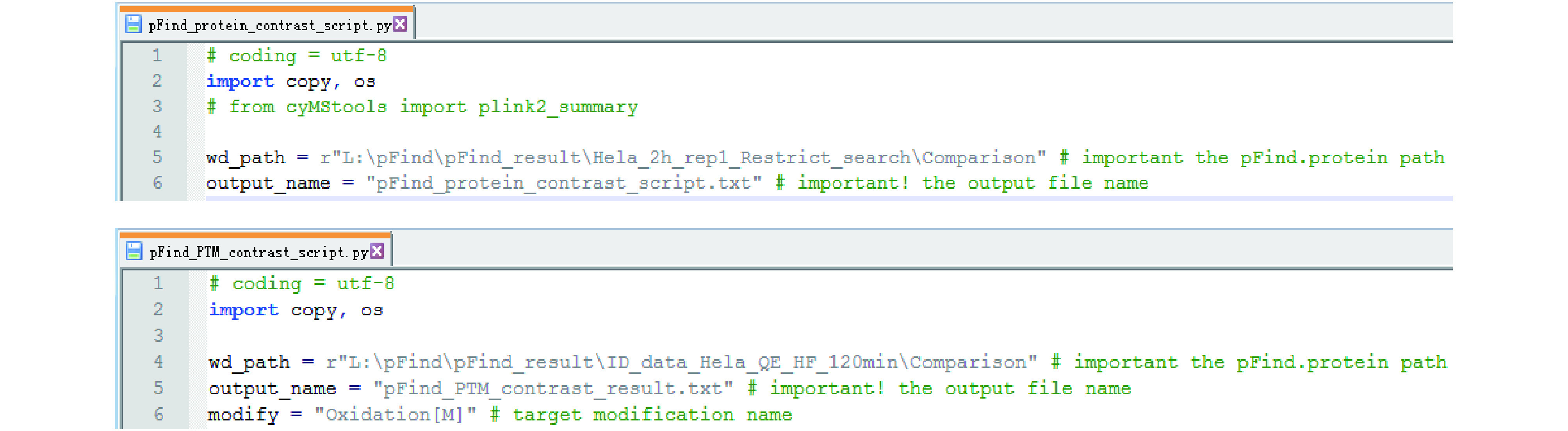
Edit “pFind_protein_contrast_script.py” (top) or “pFind_PTM_contrast_script.py” (bottom). “wd_path” is the path to the input files, “output_name” is the name of the output file, and “modify” is used to indicate a particular modification of interest

Step 20.3: Open “pFind_protein_contrast_script.py” or “pFind_PTM_contrast_script.py” using “Notepad++” or another editor (Fig. 21), specify the path of the new folder (in the sample script shown in Fig. 21, top panel, the name of the new folder is “Comparison”) and save. You may change the name of the output file if you dislike the default one. For “pFind_PTM_contrast_script.py”, one particular modification must be specified (*e.g*., “Oxidation[M]” in the bottom panel of Fig. 21).

Step 20.4: Open “Windows Explorer”, type “cmd” in the location bar, and press “Enter” to open “Command Prompt”.

Step 20.5: Lastly, run the script by entering “python pFind_protein_contrast_script.py” on the command line. Or, for PTMs: “python pFind_PTM_contrast_script.py”.

**Figure 22:**
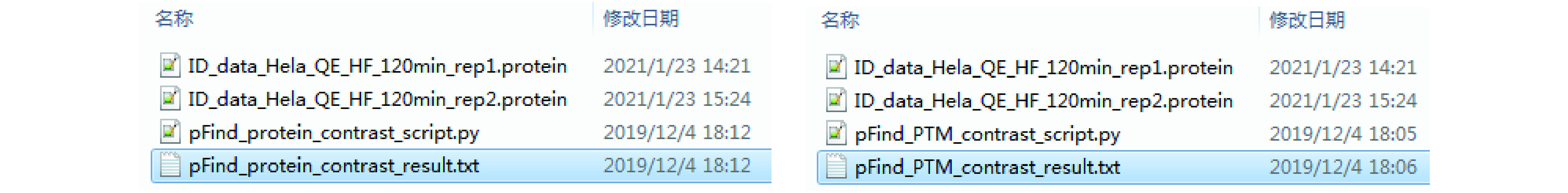
Contrast files generated to compare either proteins (left) or PTMs (right)

Step 20.6: Open with Excel the contrast file generated from the last step (Fig. 22) and explore. Header annotation of “pFind_protein_contrast_result.txt” and that of or “pFind_PTM_contrast_result.txt” can be found in supplementary Table S9 and S10, respectively.

Troubleshooting tips are listed in Table 1, and the answers to frequently asked questions (FAQ) are included in the online electronic supplementary materials.

**Table 1:** Troubleshooting

Step	Problem	Possible reasons	Solution
Step 9	pFind report “Invalid_msms_path1_path1_Path”	There are Chinese characters in RAW file path	Delete Chinese characters in RAW file path
Step 9	pFind report “Invalid_fastapath_Path”	There are Chinese characters in fasta file path	Delete Chinese characters in fasta file path
Step 16	No quantitation information	No JAVA installed or 32-bit version installed	Install JAVA https://javadl.oracle.com/webapps/download/AutoDL?BundleId=243737_61ae65e088624f5aaa0b1d2d801acb16

## Data availability

The four datasets used in this protocol, consisting of five RAW files, are described in supplementary Table S1 and S2. The datasets and the Open-pFind results have been deposited to the ProteomeXchange Consortium (http://proteomecentral.proteomexchange.org) via the iProX partner repository (Ma *et al*. 2019) with the dataset identifier PXD023901.

## How to cite this protocol

If this protocol is helpful in your work that results in a publication, we would like to ask you to kindly cite in your publication this paper and the original pFind 3 paper: Chi H, Liu C, Yang H, Zeng WF, Wu L, Zhou WJ, Wang RM, Niu XN, Ding YH, Zhang Y, *et al.* (2018) Comprehensive identification of peptides in tandem mass spectra using an efficient open search engine. Nature Biotechnology 36, 1059–1061 (PMID: 30295672; DOI: 10.1038/nbt.4236).

## Conflict of interest

Guangcan Shao, Yong Cao, Zhenlin Chen, Chao Liu, Shangtong Li, Hao Chi and Meng-Qiu Dong declare that they have no conflict of interest.

## References

[bAebersold2016] (2016). Mass-spectrometric exploration of proteome structure and function. Nature.

[bChen2020] (2020). Bioinformatics methods for mass spectrometry-based proteomics data analysis. Int J Mol Sci.

[bChi2018] (2018). Comprehensive identification of peptides in tandem mass spectra using an efficient open search engine. Nat Biotechnol.

[bCong2021] (2021). Ultrasensitive single-cell proteomics workflow identifies >1000 protein groups per mammalian cell. Chem Sci.

[bConrads2001] (2001). Quantitative analysis of bacterial and mammalian proteomes using a combination of cysteine affinity tags and 15N-metabolic labeling. Anal Chem.

[bCreasy2004] (2004). Unimod: protein modifications for mass spectrometry. Proteomics.

[bHoopmann2013] (2013). Current algorithmic solutions for peptide-based proteomics data generation and identification. Curr Opin Biotechnol.

[bHuesgen2015] (2015). LysargiNase mirrors trypsin for protein C-terminal and methylation-site identification. Nat methods.

[bMa2019] (2019). iProX: an integrated proteome resource. Nucleic Acids Res.

[bMeier2020] (2020). diaPASEF: parallel accumulation-serial fragmentation combined with data-independent acquisition. Nature methods.

[bMilo2013] (2013). What is the total number of protein molecules per cell volume? A call to rethink some published values. BioEssays.

[bMuller2020] (2020). The proteome landscape of the kingdoms of life. Nature.

[bOng2002] (2002). Stable isotope labeling by amino acids in cell culture, SILAC, as a simple and accurate approach to expression proteomics. Mol Cell Proteomics.

[bThompson2003] (2003). Tandem mass tags: a novel quantification strategy for comparative analysis of complex protein mixtures by MS/MS. Anal Chem.

[bValikangas2018] (2018). A comprehensive evaluation of popular proteomics software workflows for label-free proteome quantification and imputation. Brief Bioinform.

